# Chronic hyperpalatable diet induces impairment of hippocampal-dependent memories and alters glutamatergic and fractalkine axis signaling

**DOI:** 10.1038/s41598-023-42955-9

**Published:** 2023-09-29

**Authors:** Roberta Ribeiro, Emanuele Guimarães Silva, Felipe Caixeta Moreira, Giovanni Freitas Gomes, Gabriela Reis Cussat, Barbara Stehling Ramos Silva, Maria Carolina Machado da Silva, Heliana de Barros Fernandes, Carolina de Sena Oliveira, Leonardo de Oliveira Guarnieri, Victoria Lopes, Cláudia Natália Ferreira, Ana Maria Caetano de Faria, Tatiani Uceli Maioli, Fabíola Mara Ribeiro, Aline Silva de Miranda, Grace Schenatto Pereira Moraes, Antônio Carlos Pinheiro de Oliveira, Luciene Bruno Vieira

**Affiliations:** 1 Department of Pharmacology, ICB, Federal University of Minas Gerais, Ave. Antonio Carlos 6627, Belo Horizonte, MG CEP: 31270-901 Brazil; 2 Department of Physiology and Biophysics, ICB, University of Minas Gerais, Belo Horizonte, Brazil; 3 Department of Immunology and Biochemistry, ICB, University of Minas Gerais, Belo Horizonte, Brazil; 4 Department of Morphology, ICB, University of Minas Gerais, Belo Horizonte, Brazil; 5 Colégio Técnico, University of Minas Gerais, Belo Horizonte, Brazil; 6 Center of Research in Inflammatory Diseases, Ribeirão Preto Medical School, University of São Paulo, Ribeirão Preto, Brazil

**Keywords:** Cognitive neuroscience, Feeding behaviour, Synaptic plasticity

## Abstract

Chronic consumption of hyperpalatable and hypercaloric foods has been pointed out as a factor associated with cognitive decline and memory impairment in obesity. In this context, the integration between peripheral and central inflammation may play a significant role in the negative effects of an obesogenic environment on memory. However, little is known about how obesity-related peripheral inflammation affects specific neurotransmission systems involved with memory regulation. Here, we test the hypothesis that chronic exposure to a highly palatable diet may cause neuroinflammation, glutamatergic dysfunction, and memory impairment. For that, we exposed C57BL/6J mice to a high sugar and butter diet (HSB) for 12 weeks, and we investigated its effects on behavior, glial reactivity, blood–brain barrier permeability, pro-inflammatory features, glutamatergic alterations, plasticity, and fractalkine-CX3CR1 axis. Our results revealed that HSB diet induced a decrease in memory reconsolidation and extinction, as well as an increase in hippocampal glutamate levels. Although our data indicated a peripheral pro-inflammatory profile, we did not observe hippocampal neuroinflammatory features. Furthermore, we also observed that the HSB diet increased hippocampal fractalkine levels, a key chemokine associated with neuroprotection and inflammatory regulation. Then, we hypothesized that the elevation on glutamate levels may saturate synaptic communication, partially limiting plasticity, whereas fractalkine levels increase as a strategy to decrease glutamatergic damage.

## Introduction

Excessive consumption of highly palatable and energy-dense foods in Westernized societies is pointed out as a main environmental contributor to obesity, a pathological condition characterized by excessive accumulation of adipose tissue, as well by the imbalance between homeostatic caloric intake and energy expenditure^1^. Obesity may predispose to several harmful effects on human health, including not only metabolic and cardiovascular dysfunction, but also alterations in the central nervous system (CNS), which may affect mood, cognition, and memory^2–5^. Indeed, studies have demonstrated a negative relationship between highly palatable/energy-dense foods and obesity on different types of memory, such as episodic, aversive, and working memory^3,4,6–8^. Moreover, memory dysfunction associated with chronic consumption of highly palatable/energy-dense foods is linked to damages in decision-making, attention, executive skills, and social behavior, which may lead to a positive feedback of the obesogenic environment by maintaining food choices^3,4^. However, it is important to highlight that the complete underlying mechanism connecting the chronic consumption of highly palatable/energy-dense foods, obesity and memory deficits needs to be better addressed.

Pathological neuroinflammation is associated with negative impact on memory and cognitive function, and is closely related to metabolic alterations and peripheral inflammation^9,10^. Importantly, neuroinflammation associated with the consumption of highly palatable and energy-dense foods may be driven by peripheral low-grade inflammation through upregulation of pro-inflammatory serum levels of cytokines (IL-1β, IL-6, TNF-α, IFNγ), leptin, free fatty acids, and the reduction of blood–brain barrier permeability (BBB) tight junctions, as claudin-5 (CLDN5), occludin and zonula occludens-1 (ZO-1)^11–13^. In addition, it has been hypothesized that these mechanisms may increase the influx of peripheral immunomodulators into susceptible CNS areas, such as hypothalamus and hippocampus, intermediating the production of pro-inflammatory cytokines by glial cells, including astrocytes and microglia, which might contributes to obesity memory decline^12–15^. In this context, in chronic models of diet-induced obesity (DIO), the release of pro-inflammatory mediators by microglia, such as IL-1β, IL-6, TNF-α and nitric oxide, is associated with a decrease in neurotrophic factors production, synaptic proteins, and abnormal synaptic engulfment^6,16–19^. Furthermore, neuroinflammatory features are also associated with disruptions in neuron-glial cross-talk systems, such as the fractalkine/CX3CR1 pathway, which could lead to alterations in the number and complexity of hippocampal dendritic spines, impairment in long-term potential (LTP) formation, and cognitive decline in DIO models^16,18^. However, while data suggest a strong association between chronic consumption of highly palatable food, inflammation, and memory impairment in the context of obesity, the precise implications of this interaction on specific neurotransmitter systems remain elusive.

Glutamatergic neurotransmission has a crucial role in hippocampal memory and learning, a key area associated with the maintenance of several memory processes, such as acquisition, consolidation, memory retrieval, re-consolidation, and extinction^20–22^. In this context, glutamatergic signaling depends on the action of ionotropic (iGluR) and metabotropic receptors (mGluR), as I GluR-α-amino-3-hydroxy-5-methyl-4-isoxazolepropionic acid receptors (AMPA-R), *N*-methyl-d-aspartate receptors (NMDA-R), Kainate receptors (GluK1–GluK5) and mGluR1–mGluR8^23,24^. Moreover, dysfunctions in the regulation of glutamate levels or glutamatergic signaling are associated with disturbances in neuroplasticity mechanisms, such as the production of neurotrophic factors, memory decline, cognitive deficits, and neurotoxic events^23–26^. Although it is poorly understood, evidence pointed out that immunomodulatory event may be involved in the regulation of central glutamatergic dynamics^27–29^. For instance, data from murine hippocampal slices showed that high levels of INF-ɣ, TNF, and IL-1β are able to increase the expression, activity, and recruitment of postsynaptic AMPA and NMDA subunits (GluR1, NR2A/NR2B, respectively), suggesting some part of these cytokines in the physiopathology of glutamatergic transmission^28–30^. Moreover, a pro-inflammatory environment could induce the production of glutamate by glial cells, increasing its concentration in the synaptic cleft^27,31^. Furthermore, increased glial fibrillary acidic protein (GFAP) expression in DIO models is associated with decreased expression of astrocytic glutamatergic transporters, such as glutamate transporter-1 (GLT-1), which may be associated with neurotoxicity and neuronal plasticity damage^32^. Thus, taking into consideration that few studies have investigated the role of glutamatergic signaling in an obesogenic environment generated by highly palatable diets, regarding memory impairment^32–34^, we decided to test the hypothesis that a chronic exposure to a highly palatable/ energy-dense diet may induce hippocampal neuroinflammation, triggered by peripheral inflammation, which in turn, may lead to abnormal regulation of glutamatergic neurotransmission and memory deficits.

## Methods

### Animals and ethical aspect

C57BL/6J male mice (3–4 weeks) were purchased from the animal facility of Universidade Federal de Minas Gerais (UFMG). Animals were housed five per cage under the following environmental conditions: 12 h light/12 h dark cycle, temperature at 24 ± 2 °C with food and water provided ad libitum. The sample size for the animal experiments was determined through a rigorous calculation, considering the variance observed in specific assays, and was approved by the institutional animal use ethics committee (CEUA-UFMG no. 217/2020). All experiments were carried out in accordance with ARRIVE guidelines (https://arriveguidelines.org). All methods were performed in accordance with the relevant guidelines and regulations.

### Diets and experimental design

After 1 week of habituation, mice were divided into two groups, and fed for 12 weeks with an isocaloric diet (American Institute of Nutrition 93-Growth diet (AIN93G, (carbohydrates: 64%, proteins: 20%; lipids: 16%; 3.9 kcal/g) or a highly palatable/energy-dense diet [high butter and sugar diet (HSB), (carbohydrate: 36%, proteins: 16%; lipids: 48%; 4.9 kcal/g)]^11^ (Supplementary data, Fig. S1A). Mice were weighed once a week. In addition, the cumulative energy intake was calculated by summing weekly values of the food amount eaten per cage multiplied by the caloric value of each diet^35^. Between the 11th and 12th week of the diet-induced obesity protocol, behavioral analyses were performed to evaluate memory and learning deficits (n = 10 per group) (Supplementary data, Fig. S1B). In parallel, a separate cohort of animals was submitted to the oral glucose tolerance test (n = 10 per group) (Supplementary data, Fig. S1A). At the end of week 12th euthanasia was performed in order to obtain tissues for biochemical, molecular and histological assays (n = 5–10 per group) (Supplementary data, Fig. S1B). Importantly, with the exception of histological analyses, all euthanasia was performed by decapitation to avoid interference with basal glutamatergic signaling. Immediately after euthanasia, the brain was isolated and the hippocampus was dissected for molecular measurements. Regarding serum analyses, cervical blood samples were collected in heparinized tubes, centrifuged at 1500×*g*, 4 °C, for 15 min, and stocked at − 70 °C until use. In addition, topics 2.6 and 2.9 contain information on the euthanasia and tissue preparation for histological and hippocampal synaptosome assays, respectively.

### Behavioral assessment

All behavioral tests were performed during the light cycle, under controlled conditions of temperature and brightness (24 ± 2 °C, 70–80 lx). Furthermore, 30 min acclimation period was given to mice before starting the experiments. All apparatus were cleaned with 70% ethanol between the experimental sessions to avoid olfactory bias. None of mice cohorts used were subjected to more than two different behavioral tests (Supplementary data, Fig. S1B). Experimental tasks were designed to consider the most stressful behavioral experiment to be performed at last. In addition, one to four days of interval between experimental tasks were applied (Supplementary data, Fig. S1B). Also, mice were not deprived of food or water during experimentation. All behavioral memory tasks were manually analyzed with the support of the software X-Plot-Rat ^®^2005 (developed by the research team of Dr. Morato, Faculdade de Filosofia, Ciências e Letras de Ribeirão Preto, USP, Brazil).

#### Open field

To evaluate locomotor and exploratory behavior, the open field test was performed. Mice were randomly placed in a cylindrical and circular acrylic apparatus (30 cm × 40 cm), where free exploration was allowed for 30 min. Total distance traveled (cm), the average speed (m/s), as well as the time spent in the center of the arena (s) were recorded and analyzed by Any-maze Video Tracking System Software (version 5.26, Stoelting^®^).

#### Y maze test

The evaluation of short-term memory was performed by using the Y- maze test. For that, a grey plastic maze with three arms (A, B, C) (30 cm length, 6 cm width, 20 cm height) arranged at a 60° angle was used. In addition, different visual clues were used in each arm to help the mice to recognize them. Mice were placed individually at the distal part of A-arm and free to explore the apparatus for 8 min. An arm entry was considered only when both hind paws were placed completely inside. Percent alternation was calculated using the following formula:

$$({\text{Number of alternations}}/[{\text{Total number of arm entries}} - {2}])\, \times \,{1}00.$$

#### Object location test (OLT) and object recognition test (ORT)

A white square acrylic open field (38 × 38 × 15 cm) was utilized for both tests. At the first day, mice were habituated to the apparatus for 5 min. 24 and 48 h after habituation, mice were re-exposed to the open field containing two identical objects, two identical plastic bottles (20.5 cm high, 6 cm wide), colored red and filled with 11 cm of water. In addition, these objects were located symmetrically at 80 mm of the apparatus wall and vertically separated from each other by 6 cm. Free exploration was measured for 10 and 5 min, in these respective days. To the OLT and ORT test (72 h after habituation), one of the objects was displaced 6 cm away from the original position (novel position) or one of the objects was replaced by a new object, without altering the original localization. In addition, the new object consisted of multi-colored lego blocks, stacked vertically to have the same dimensions as the familiar object. In both tests, mice were allowed to freely explore for 5 min. Interaction with the objects was defined as sniffing until 2 cm of distance from the object, as well as touching the objects with the nose or forepaws. The percent of total investigated time of the new object or moved object was calculated according to the following formulas^36^:

$${\text{OLT }}\left( \% \right): \, [{\text{Time}}_{{\text{with novel object localization}}} /{\text{Time}}_{{\text{with novel object localization}}} + {\text{ Time}}_{{\text{with familiar object localization}}} ]\, \times \,{1}00;$$

$${\text{ORT }}\left( \% \right): \, [{\text{Time}}_{{\text{with novel object}}} /{\text{Time}}_{{\text{with novel object}}} + {\text{ Time}}_{{\text{with familiar object}}} ]\, \times \,{1}00.$$

#### Contextual fear conditioning

A conditioning chamber (330 × 200 × 300 mm^3^) with a grid floor, and transparent acrylic board customized with black vertical lines was used for mice contextual fear conditioning^37^. The first day of the experimental procedure consisted of an induction of aversive conditioning to the context. For this, mice were individually habituated within 120 s to the apparatus and subsequently subjected to a foot shock (Unconditioned stimulus (US): 0.7 mA per 2 s). After the first shock, an interval of 60 s was given before a new US was applied. In total, three US were performed with a 60 s interval between them. At the end of the session, animals were removed from the apparatus and returned to their cages. 24 h after the aversive conditioning, the evocation memory test was executed in order to evaluate the memory retrieval. For this, mice were re-exposed to conditioning context, without receiving new shocks, for 5 min. Furthermore, in order to check for possible alternations in memory extinction process, we induced fear memory extinction by prolonging the exposition of the mice to the context for more than 15 min (totaling a 20 min of test). Finally, on the third day, mice were re-exposed to context, for 5 min, for the evaluation of memory extinction (memory extinction test). For analyses, freezing was considered as the entire absence of movement, excluding breathing periods. The computation of freezing was performed every minute during the entire memory evocation and extinction test session. All results were expressed in percent of freezing.

#### Social interaction and social memory test

The evaluation of social interaction and social memory was conducted in a transparent and rectangular acrylic box (60 × 40 cm) divided into three compartments (20 × 40 × 22 cm) separated by two interior doors. Adult mice were individually habituated for 5 min in the central compartment. Subsequently, for the evaluation of social interaction, a plastic transparent cylinder (10 cm diameter with distributed holes) containing an unfamiliar male juvenile mouse (Stranger 1) was introduced into one of the chambers and an identical empty plastic cylinder was introduced in the remaining chamber. After, inner doors were opened and mice were free to explore the social apparatus for 10 min. Additionally, the location chamber with the juvenile mouse or the empty cylinder was alternated between experimental sessions. Then, a second unfamiliar mouse (Stranger 2) was placed in the empty cylinder for the evaluation of social memory. Thus, the adult mouse was free to explore the apparatus for another 10 min. Furthermore, in order to assess short- and long-term social memory, an additional single-chamber protocol was performed^38^. In summary, the training session consisted of habituating of mice for 15 min in a standard cage. After, a plastic transparent cylinder containing an unfamiliar male juvenile mouse was presented to an adult mouse for 5 min. For the evaluation of short-term memory (STM) and long-term memory (LTM), the same experimental procedures, and the same juvenile mouse, were re-exposed to the adults’ mice after an hour and a half and 24 h after the training session, respectively. Importantly, in three and one chamber paradigms, none of the juvenile mice were used more than four times. Moreover, in both protocols the social investigation was computed at the time that the resident mice introduced their nose or whiskers inside any of the cylinder's holes^38^. The social interaction and social memory index were calculated according to the following formulas:

$${\text{Social interaction }}\left( \% \right): \, [{\text{Time}}_{{\text{with stranger 1}}} /{\text{Time}}_{{\text{with stranger 1}}} + {\text{ Time}}_{{\text{with empty object}}} ]\, \times \,{1}00;$$

$${\text{Social memory }}\left( \% \right): \, [{\text{Time}}_{{\text{with stranger 2}}} /{\text{Time}}_{{\text{with stranger 2}}} + {\text{ Time}}_{{\text{with stranger1}}} ]\, \times \,{1}00.$$

STM and LTM were expressed by the raw interaction time with juvenile mice as compared to training, as well by the percentage of time of interaction with the juvenile mice considering the total time tested^38^.

### Oral glucose tolerance test

The oral glucose tolerance test (oGTT) was adapted from Pedro et al. (2020)^39^. At 12th week, a separated cohort of mice was submitted to fast for 6 h (07 AM to 01 PM). Glucose (30%) was administered by gavage (2 g/kg). Blood sampling was obtained by the tip of the tail, using a scissor, and glucose concentration (mg/dL) was measured with a glucometer (On call plus II^®^), before glucose gavage, and 15, 30, 60, 90, and 120 min thereafter. The area under the curve (AUC) was calculated to assess differences in glucose metabolism between the groups of interest^40^.

### Cholesterol measurement, adiposity and lee index

The total cholesterol levels were measured in the serum of mice by a commercial colorimetric enzymatic test in accordance with manufacturer instructions (Bioclin, MG, Brazil). A spectrophotometer with wavelength of 500 nm was used to acquire data. The following formula was utilized for final concentration determination: total cholesterol (mg/dL): [sample absorbance × 200]/standard absorbance. Alterations in body composition caused by HSB diet and cardiometabolic risk were evaluated through adiposity measurements and Lee Indexes. The sum of the weight of visceral adipose tissue fat pads (VAT) compounds from the epididymal (EAT), mesenteric (MAT), and retroperitoneal (RPAT) fat pad was used for the calculation of visceral adipose tissue index: [VAT (g)/body weight (g)] × 100. Lee index was calculated by the following formula:

$$[{\text{body weight }}\left( {{\text{g}}^{{0.{\text{33}}}} } \right)/{\text{naso-anal length }}\left( {{\text{cm}}} \right)].$$

### Intracardiac perfusion and brain slices

At 12th week, animals were anesthetized with ketamine (80 mg/kg, i.p) and xylazine (8 mg/kg, i.p), and then submitted to a thoracotomy for heart exposition. A needle coupled to a peristaltic pump infusion system was inserted in the left ventricle and a small incision was made in the right atrium, allowing the withdrawal of blood by infusion of phosphate saline buffer (pH 7.4; 5 ml/min; 30–100 ml per animal). After animals were perfused with a paraformaldehyde solution (PFA 4%, pH 7.4, 20–30 ml per animal), decapitated, and their brains were stored overnight in PFA 4%. Subsequently, brains were cryoprotected, before being frozen in ice isopentane (99%, 20 s), by gradual dehydration in a 15% sucrose buffer (pH 7.4), and then in a 30% sucrose buffer (pH 7.4,) until complete precipitation. Brains were sliced into 30 µm sections in a cryostat at −25 °C (Leica Biosystems, Buffalo Grove, USA).

### Evaluation of microglial density and morphology

Microglial morphological features may provide information about cellular functional phenotypes^41^. Thus, in order to evaluate whether an obesogenic diet affects the microglial densitometry and morphological parameters, immunohistochemistry analyses were performed. Slices were incubated with citrate buffer (pH 6.0, 70 °C, 1 h), washed with TBS (3×, 5 min), pretreated with 1% of H_2_O_2_ (15 min), washed (TBS, 3×, 5 min) and blocked with BSA 4% in TBS triton 0.5% for 1 h at room temperature followed by primary antibody incubation (rabbit anti-Iba-1 (1:500; Wako Chemicals, Osaka, Japan) for 48 h at 4 °C. After washing (TBS, 3×, 5 min), slices were incubated with biotinylated secondary antibody (goat anti-rabbit (1:500 in TBS; Vector Laboratories, Burlingame, USA; overnight at 4 °C). Posteriorly, slices were incubated with Avidin–Biotin solution (ABC) (VECTASTAIN ABC kit, Vector Laboratories, Burlingame, USA) for 1 h. For revealing the staining, DAB (Sigma-Aldrich, St. Louis, USA) diluted in TBS and activated with H_2_O_2_ 0.04% was utilized in accordance with manufactured instructions. Sections were washed (TBS, 3×, 5 min) and mounted in histological slices. After drying, slices were sequentially dehydrated in ethanol 70, 80, 95% (3 min), and 100% (two twice, 4 min), and cleaned in xylene (two times, 5 min) before mount cover slices with DPX (Sigma-Aldrich, St. Louis, USA). The images of DG, CA1, CA2-CA3 were acquired in a light microscopy (APOTOME.2, Carl Zeiss, Jena, Germany) in 20× and 40× magnification, for densitometry and morphological analysis, respectively. The quantification of optical density and measurement of cell body to cell size ratio was performed using FIJI software (NIH, Bethesda MD, USA) in accordance with previously published methods^41,42^. For this, three brain slices per animal (technical replicates), six photos for DG, CA1 and 3 pictures for CA2–3 were used for analysis.

### Evaluation of astrocyte reactivity

Immunofluorescence analyses were performed to evaluate astrocyte reactivity. Hippocampal brain slices were incubated with citrate buffer (pH 6.0) at 70 °C for 60 min, washed with TBS (3×, 5 min), and blocked overnight with 4% BSA in 0.5% TBS triton (TBSt), before incubation with primary antibody (mouse anti-GFAP (1:500; Millipore, Darmstadt, Germany) for 48 h at 4 °C. Afterwards, slices were washed with TBS (3×, 5 min) and incubated, at dark, with secondary antibodies (120 min), (goat anti-mouse, 1:1000; Alexa Fluor 488, Life Technologies, Carlsbad, USA). Finally, after the washing (TBS, 3×, 5 min), 1 µg/ml of DAPI was added (30 min). Sections were washed (TBS, 3×, 5 min), mounted in histological slices, and after drying, mounted cover with Fluoromount (Sigma-Aldrich, St. Louis, USA). Images were acquired with a fluorescence microscopy (AxioCam M2, Carl Zeiss, Jena, Germany) in 20× magnification. The quantification of cell bodies was performed in the dentate gyrus (DG), CA1, CA2–CA3 using FIJI software (NIH, Bethesda MD, USA). For this, two brain slices per animal (technical replicates), four photos for DG, CA1 and two pictures for CA2–3 were used for analysis. All results were represented as percent of the area stained for GFAP and the mean fluorescence intensity of the cells.

### Measurement of cortical and hippocampal glutamate release

#### Purification of synaptosomes

The hippocampal pools of synaptosomes (3 mice per pool) were obtained in accordance with Dunkley et al. (1986)^43^. After decapitation and dissection, tissues were immediately homogenized in an ice gradient solution (320 mM sucrose, 0.25 mM dithiothreitol, 1 mM EDTA), centrifuged (1000*g*, 10 min, 4 °C) and the resultant supernatant was purified by separation in a discontinuous percoll gradient (Sigma Aldrich^®^) at concentrations of 23%, 15%, 10%, and 3% (pH 7.4). After centrifugation (6000*g*, 15 min, 4 °C), the isolated synaptosome was re-suspended in a Krebs–Ringer–HEPES solution (KRH) without CaCl_2_ (124 mM NaCl, 4 mM KCl, 1.2 mM MgSO_4_, 10 mM glucose, 25 mM HEPES, pH 7.4, 10 mg/ml), and centrifuged (6000*g*, 15 min, 4 °C). The Pellet was obtained, centrifuged (3.333*g*, 60 s), re-suspended in KRH, and incubated at 37 °C per 30 min. This process was repeated once more and synaptosome aliquots were immediately led for reading and quantification.

#### Measurements of glutamate release

Glutamate release was indirectly quantified by a fluorescent method^44^. Fluorescence quantification was measured using a fluorimeter (Synergy TM2, Biotek^®^). Excitation and emission wavelengths were recorded at 360 nm and 450 nm, respectively. Isolated nerve terminals were incubated with CaCl2 (1 mM), and NADP+ (1 mM) in KRH medium (2 min). Glutamate dehydrogenase (Sigma Aldrich^®^; 50 units per well) was added to each well, and readings were performed until the fluorescence reached balance (5 min). KCL 33 mM was used as a depolarizing stimulus (10 min). Finally, calibration curves were obtained after standard amount of glutamate (Sigma Aldrich^®^; 5 nM/μL) was added to the medium (5 min). Glutamate levels were normalized to the total amount of synaptosomal protein that was obtained through Bradford assay.

### Real-time PCR

RNA was isolated from the hippocampal samples with TRIZOL reagent^®^ (Invitrogen, Burlington, EUA) in accordance with manufacturer instructions. The RNA concentration and quality were determined by spectrophotometer lecture (NanoDropTM (Thermo Scientific, Wilmington, USA). cDNA was prepared from 500 ng of the isolate RNA through reverse transcriptase reaction using 300 ng of random primers. Quantitative PCR was performed with specifc primers and Power SYBR^®^ Green PCR Master Mix kit (Thermo Scientific, Wilmington, USA) in accordance with manufacturer instructions and QuantStudio™ 7 Flex Real-Time PCR System were employed for execution of the qPCR, as well as data analysis. Specific primers sequence of the target genes (AMPA: GLUR2; NMDA: NR1, NR2A, NR2B; mGluR5: Grim5, GLT-1:SCLA2), BBB proteins [Claudin-5, Occludin, Zoocludin-1(ZO-1)], cytokines and chemokines (IL-1β, IL-6, TNF-α, INF-ɣ, CX3CL1, and CX3CR1), as well synaptic plasticity factors [Synaptophysin: SYP, postsynaptic density protein 95 (PSD-95): DLG4, and activity-regulated cytoskeleton-associated protein (ARC)] were designed with Primer3plus software and validated with NCBI Primer-BLAST (Supplementary data, Table S1). Samples were prepared in triplicate and gene expression variations were determined by the ΔCt method using the RPL32, as a constitutive normalizing gene. In the case of cytokines, the mean of two normalizing genes was used by the ΔCt calculation: GAPDH and HPRT.

### Evaluation of neurotrophic factors, fractalkine, and cytokine levels

In order to measure the levels of neurotrophic factors (BDNF, NGF, GDNF), fractalkine (CX3CL1), serum leptin, and inflammatory cytokines (IL-1β, IL-6, TNF-α, INF-ɣ), in the serum and hippocampus, an ELISA assay was performed. An cold extraction solution (100 mg of tissue per milliliter), containing Tris–HCl (20 mM); NaCl (137 mM); NP40 (1%); Glycerol (10%); phenyl methyl sulfonylfluoride (1 mM) or aprotinin A (0.5 µg/mL), Pesptatin A (1 μM), EDTA (10 mM), E-64 (10 μM), sodium vanadate (0.5 mM), and deionized water was added to hippocampus samples. Samples were mechanically processed, and centrifuged (14,000 rpm, 4 °C, 20 min). The hippocampal supernatant and serum samples were stocked at −80 °C until use. The levels of all interesting targets were measured using commercial kits from R&D system (DuoSet, Minneapolis, MN) in accordance with manufacturer instructions. Results were acquired on a spectrophotometer and expressed as pg/mL or pg/100 mg of tissue.

### Statistical analysis

Statistical analyses were performed using the graphpad prism software 8 (San Diego, USA). The normality and homoscedasticity of the data were tested using Shapiro–Wilk and Levene's test, respectively. Outliers were detected via the box-plot interpolation method. Data were expressed by mean ± SEM. Furthermore, as appropriated, unpaired test-t, Mann–Whitney test, repeated measures ANOVA, two-way ANOVA, and mixed effect model, followed by Tukey post hoc test were employed. In addition, in ORT and OLT we also used a one-sample T-test for comparing the experimental control means with a hypothetical mean of 0.5 (different from random)^45^. The level of statistical significance adopted was p < 0.05.

##  Results

### HSB diet induces obesogenic phenotype and metabolic abnormalities

Our results showed a significant time × diet interaction effect, associated with a gradual increase of body weight in our DIO model (two-way ANOVA, F_11, 456_ = 15, 42, p < 0.0001) (Supplementary data, Fig. S2A, B). Furthermore, the HSB group presented a higher adiposity index (unpaired T student-test, t_18_ = 5.832, p < 0.0001), increased lee index (unpaired T student-test, t_18_ = 2.330, p = 0.0316), and elevated cumulative energy intake (unpaired T student-test, t_10_ = 7.639, p < 0.0001) (Supplementary data, Fig. S2C–F), all pointing out to a possible alteration in body composition caused by the increase of palatable diet consumption. Regarding metabolic dysfunction, a chronic exposure to HSB diet induced higher fasting glucose levels (unpaired T student-test, t_18_ = 3.548, p = 0.0023) (Supplementary data, Fig. S2G), and increased oral glucose intolerance (unpaired T student-test, area under curve (AUC) t_18_ = 3.117, p = 0.0052) (Supplementary data, Fig. S2H–I). Additionally, elevated levels of total cholesterol were observed (Mann–Whitney test, total cholesterol, p = 0.0002) (Supplementary data, Fig. S2J). Taken together, these data suggest that the HSB diet was effective in inducing obesity features, which is in accordance with previous literature^11^.

### HSB diet impairs the process of memory reconsolidation and extinction

**Figure 1:**
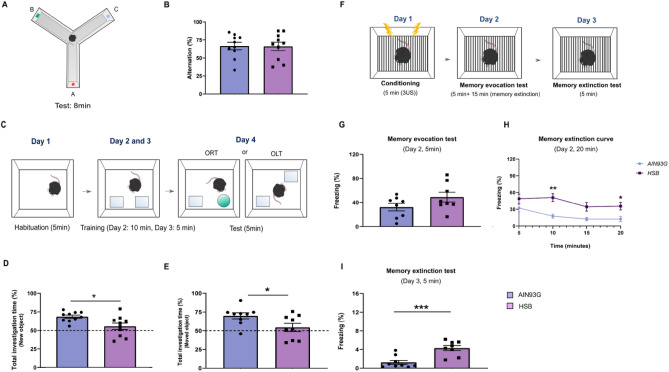
Chronic consumption of HSB diet does not alter operational short-term memory behavior, but induces alterations in reconsolidation and extinction of memory. **(A)** Schematic representation of method used in the Y maze test. **(B)** Evaluation of operational short-term memory by the Y maze test. The figure represents the calculation of arms alternation index (%) in the apparatus. (**C**) Representations of the novel object recognition and novel object localization test (ORT and OLT) protocol. (**D,E**) Measurement of total investigation time index (%) for ORT and OLT tests, respectively. (**F**) Schematic representation of protocol used in contextual conditioned fear. (**G**) Evaluation of memory evocation during the first 5 min of re-exposure to the context. **(H)** Representative curve of extinction during the entire re-exposure of the context memory evocation session. **(I)** Measurement of memory extinction, 24 h after the re-exposure session, during a 5 min duration session. Error bars represent the mean ± SEM; n = 9 (**E**,**G–I**), and 10 (**B**,**D**). Unpaired two tail T-student test (**B,D,E,G,I**), and Two-way ANOVA followed by Tukey’s post-test (**H**). *p < 0.05; **p < 0.01; ***p < 0.001.

To investigate possible memory impairment in our DIO model, we first assessed whether the chronic exposure to an obesogenic diet was able to induce changes in the exploratory behavior, and short-term memory. For that, the open field and Y maze test were performed, respectively (Supplementary data, Fig. S3 and Fig. 1A). We observed that in both behavioral tests, none of the investigated parameters were affected by the HSB diet (Open field: unpaired T student-test, Total traveled distance t_16_ = 0.4205, p = 0.6797, mean speed t_16_ = 0.4873, p = 0.6327; Mann–Whitney test, time in center, p = 0.1359. Y maze: unpaired T student-test, alternation index t_18_ = 0.06189, p = 0.9513) (Supplementary data, Fig. S3, and Fig. 1B). Next, in order to evaluate the impact of HSB diet in events associated with hippocampus-dependent long-term memories, mice were subjected to the ORT, OLT, or contextual fear conditioning (Fig. 1C, F). Notably, HSB diet was able to reduce the total investigation time in ORT and OLT, which may suggest a decline in episodic memory (Unpaired t-test, Total investigation time: ORT t_18_ = 2.731, p = 0.0137; and OLT t_16_ = 2.315, p = 0.0342) (Fig. 1D, E). Although memory retrieval was similar in both groups (unpaired t-test: memory evocation test, t_14_ = 1.612, p = 0.1292) (Fig. 1G), we observed that the obese group presented an abnormal extinction curve, besides an increased freezing percentage in the extinction test as compared to the lean group (two-way ANOVA, memory extinction curve: F_1, 56_ = 30.06, p < 0.0001; unpaired t-test: memory extinction test, t_15_ = 4.417, p = 0.005) (Fig. 1H, I). Therefore, this dataset may suggest that HSB diet is able to impact specific memory events, such as extinction. Finally, to determine whether the long-term memory impairment promoted by the HSB diet may impact social skills, we conducted some assays to verify social interaction and social memory (Supplementary data, Fig. S4A, D). First, our preliminary data showed that HSB diet was not able to decrease social interaction and social memory index (Mann–Whitney test, social interaction index, p = 0.1481; unpaired t-test, social memory index t_18_ = 0.3997, p = 0.6941) (Supplementary data, Fig. S4B, C). Furthermore, in the single chamber social interaction test, we observed that similarly to the lean group, the HSB group showed a decrease in the raw interaction time with familiar juvenile rats in the STM, and a tendency to decrease the interaction in the LTM assessment tests (one-way repeated measure, STM, p = 0.0094; LTM, p = 0.0678) (Supplementary data, Fig. S4E). Interestingly, this pattern was also corroborated with the percentage of STM and LTM, that remained unchanged in the HSB group, suggesting that social skills and social memory were not affected by the obesogenic environment (unpaired t-test, STM index t_16_ = 0.08577, p = 0.9327; LTM index t_16_ = 1.378, p = 0.1872) (Supplementary data, Fig. S4F, G).

### Hippocampal neuroinflammation is not associated with chronic consumption of HSB diet

**Figure 2:**
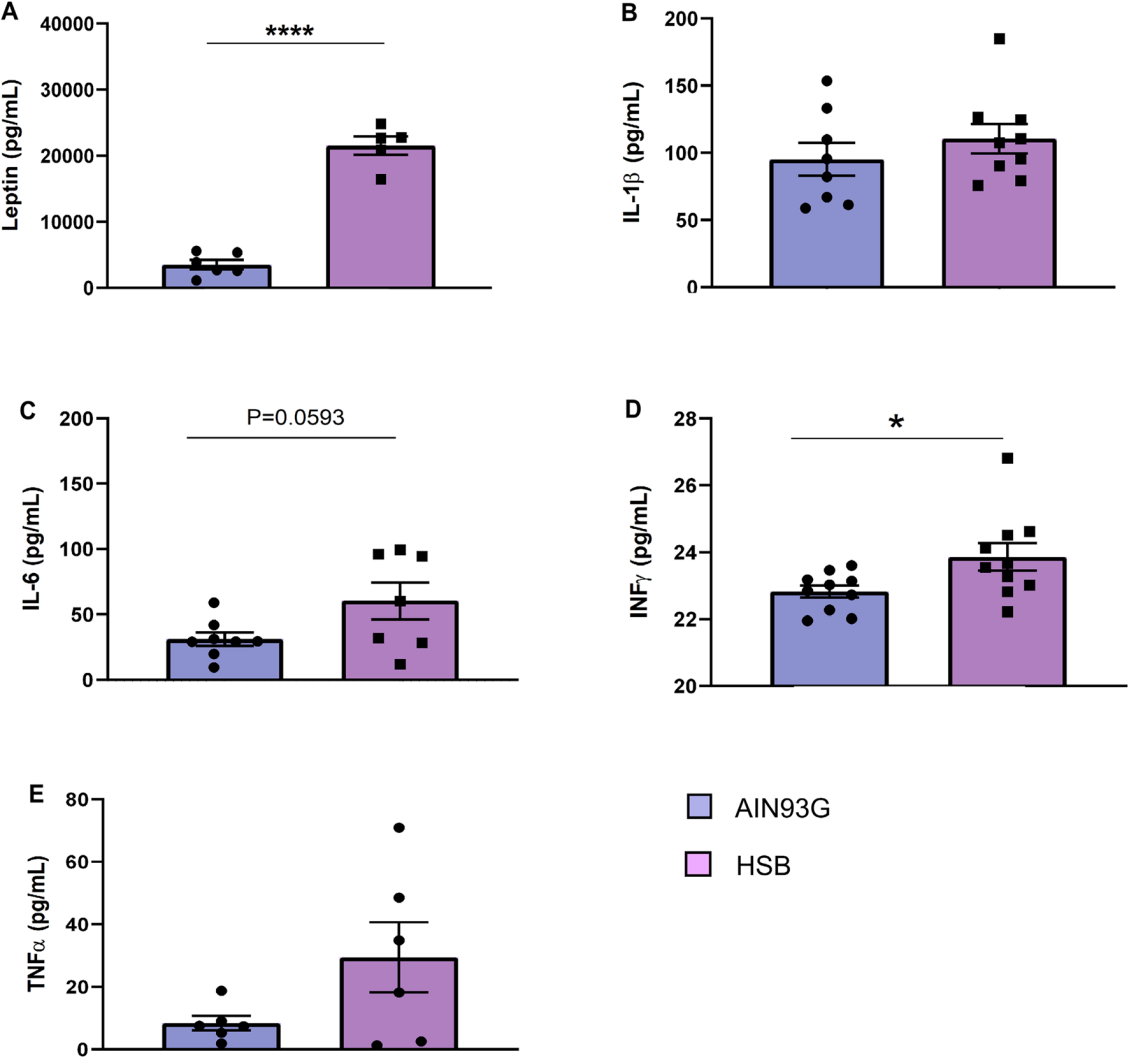
Chronic consumption of HSB induces a peripheral pro-inflammatory environment. **(A–E)** Measurements of the protein levels of serum leptin (**A**), IL-1β (**B**), IL-6 (**C**), INF-ɣ (**D**), and TNF-α (**E**). Error bars represent the mean ± SEM; n = 5–6 (**A,E**); 7–9 (**B,D**), and 10 (**D**). Unpaired two tail T-student test. *p < 0.05, ****p < 0.0001.

**Figure 3:**
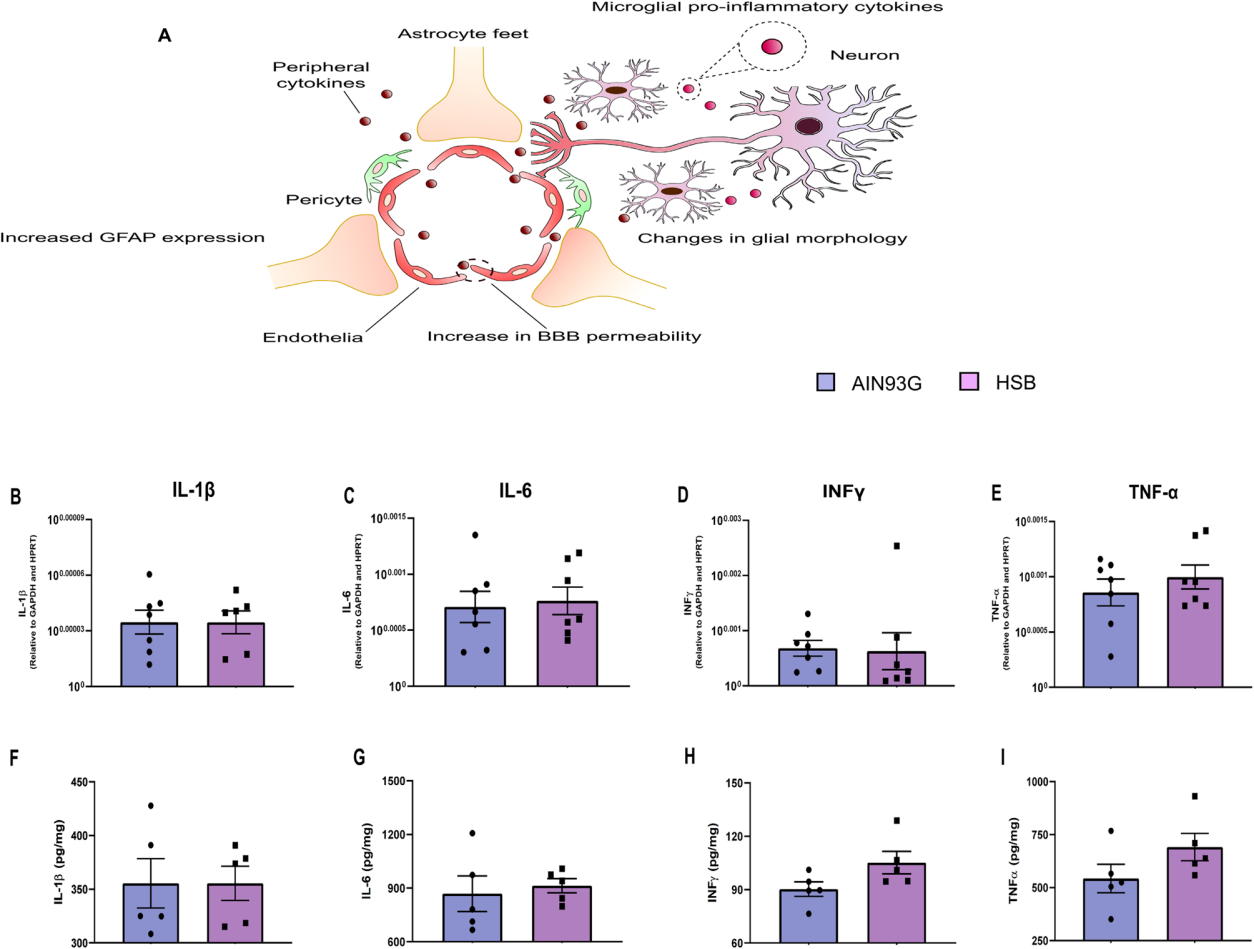
Chronic consumption of HSB diet is not able to modulate cytokine levels in the hippocampus. **(A)** Schematic representation of postulated mechanism associated with BBB breakdown, due to the presence of proinflammatory peripheral cytokines, and neuroinflammation establishment in an obesity condition. (**B–E**) Quantification of pro-inflammatory cytokine mRNA levels (IL-1β, IL-6, INF-ɣ, and TNF-α). (**F–I**) Measurements of protein levels of IL-1β, IL-6, INF-ɣ, and TNF-α. Error bars represent the mean ± SEM; n = 7 (**B–E**), and n = 5 (**F–I**). Unpaired two tail T-student test (**B,D,E–I)** and Mann–Whitney test (**C**).

**Figure 4:**
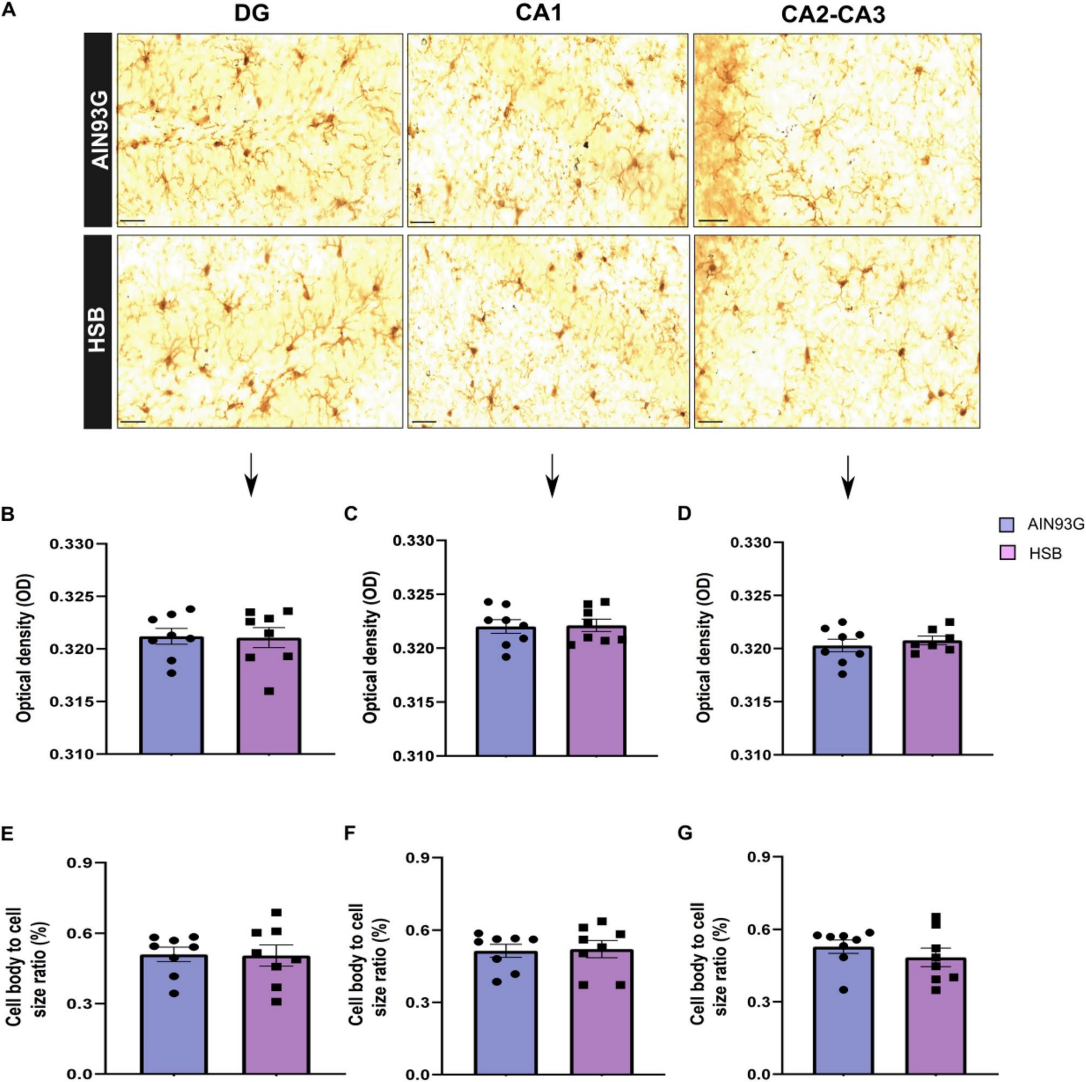
Chronic consumption of HSB diet did not alter densitometry and morphological parameters in hippocampal microglia. **(A)** Representative photomicrography of Iba-1 stained cells in dentate gyrus (DG), CA1, and CA2-3 of lean (AIN93G) and obese (HSB) groups. **(B–D)** Mensuration of microglial optical densitometry of in DG (**B**), CA1 (**C**), and CA2–3 (**D**). **(E–G)** Cell body to cell size ratio of microglia in DG (**E**), CA1 (**F**), and CA2–3 (**G**). Microscope lens ×40 and 50 µm scale bar for the images. Error bars represent the mean ± SEM; n = 8. Unpaired two tail T-student test (**B–E**), and Mann–Whitney test (**F,G**).

**Figure 5:**
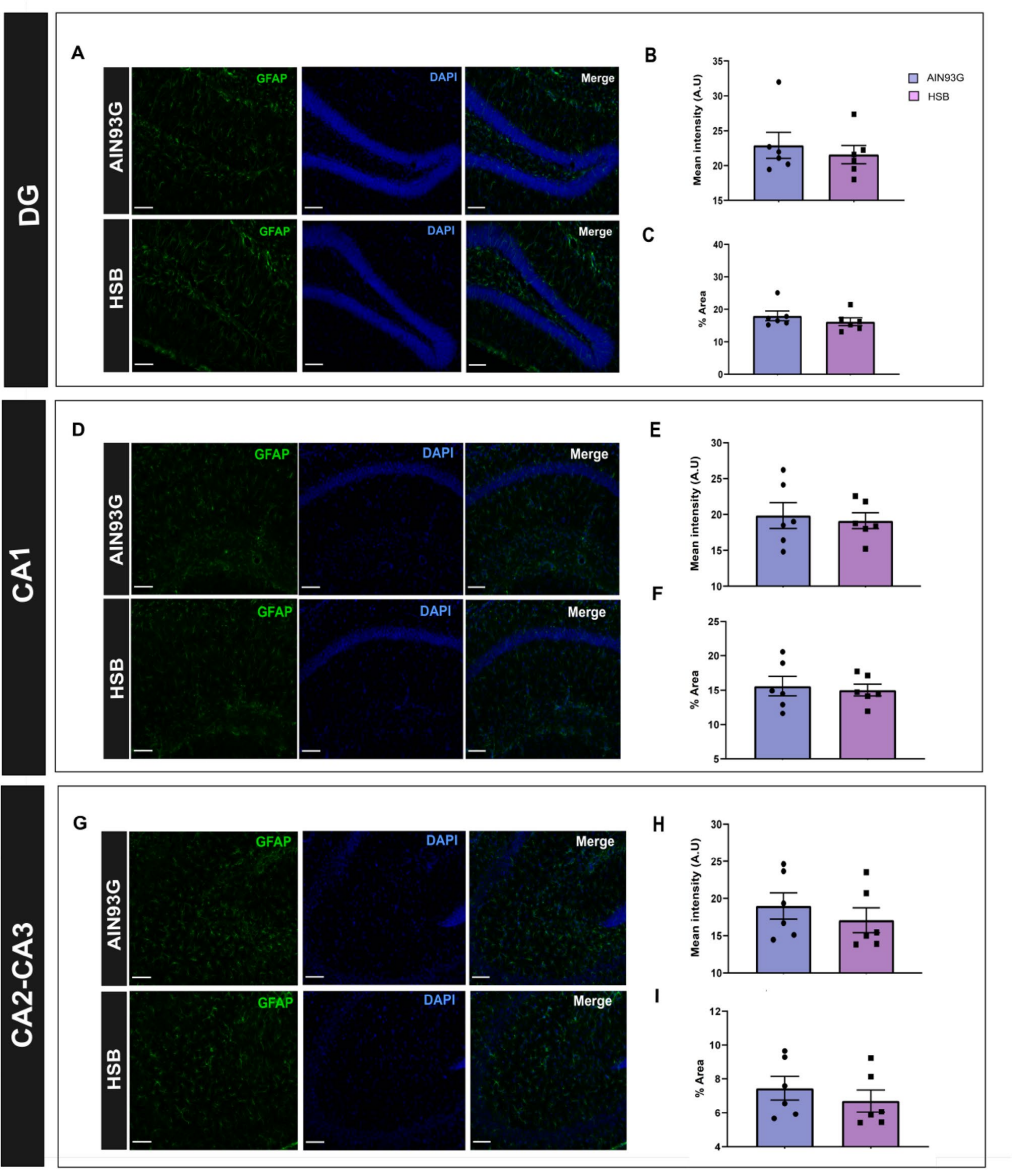
HSB diet does not alter hippocampal astrocyte reactivity. **(A,D,G)** Representative photomicrography of GFAP positive cells in the dentate gyrus (DG), CA1, and CA2–3. **(B,E,H)** Mean intensity fluorescence of GFAP stained for GFAP+ cells in DG, CA1, and CA2-3. (**C,F,I)** Percentual area stained for GFAP+ cells in DG, CA1, and CA2–3. Microscope lens ×20  and 80 µm scale bar for the image representation. Error bars represent the mean ± SEM; n = 6. Mann–Whitney test (**B,C**), and unpaired two tail T-student test (**E,F,H,I**).

**Figure 6:**
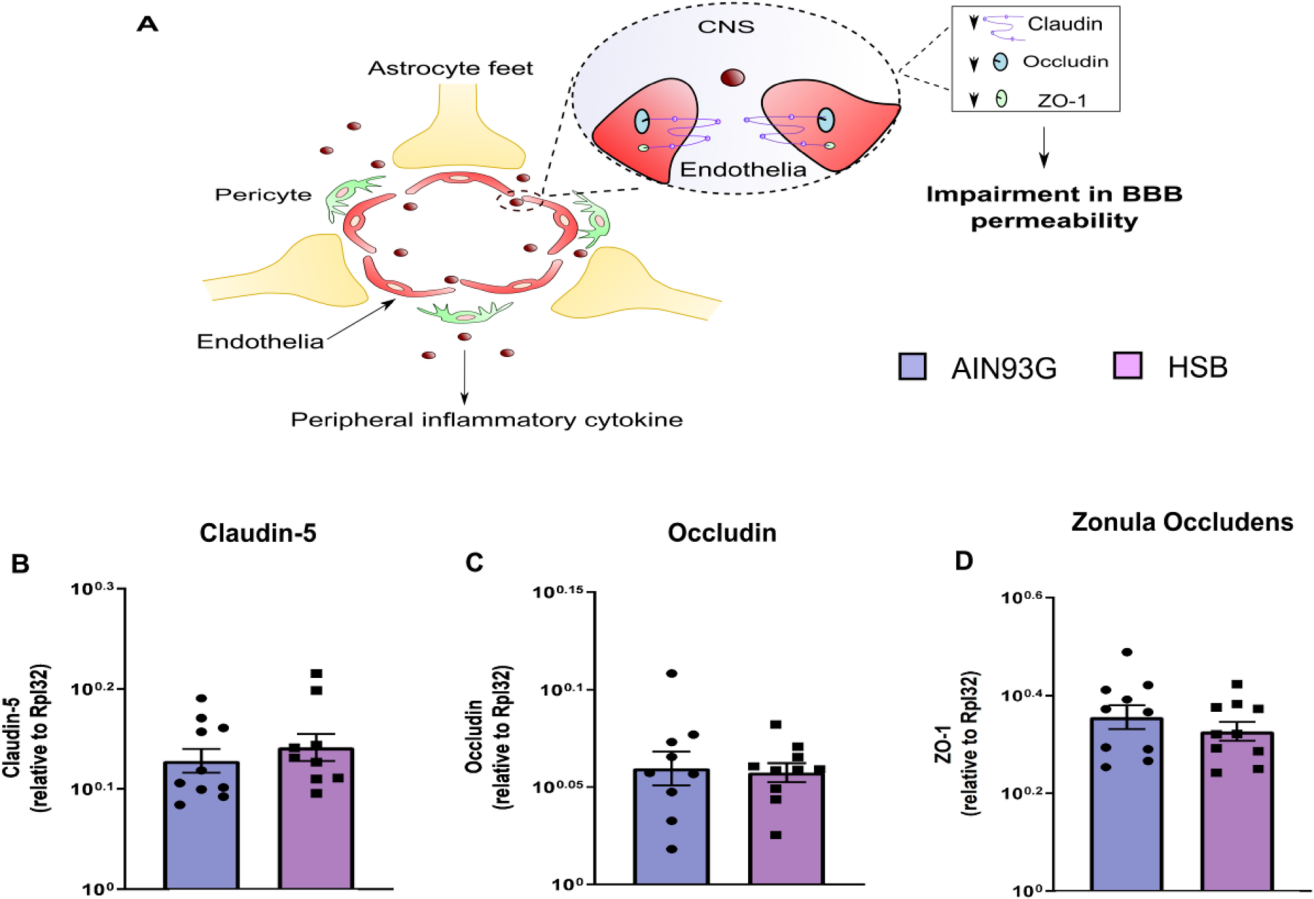
Chronic consumption of HSB diet is not able to decrease the expression of hippocampal blood–brain barrier (BBB) tight junctions. **(A)** Schematic representation of BBB increases permeability associated with the decrease of tight junctions due to a chronic peripheral inflammation environment. **(B–D)** Quantification of hippocampal Claudin-5, Occludin, and Zonula occludens-1 (ZO-1) mRNA levels. Error bars represent the mean ± SEM; n = 9–10. Unpaired two tail T-student test.

Considering the hypothesis that an increased peripheral inflammatory environment may impair BBB permeability, contributing to glial reactivity, and consequently to memory decline, through neuroinflammatory mechanisms^12^, we decided to investigate whether obesity induced by HSB diet could modulate serum leptin levels, likewise the pattern of peripheral and hippocampal pro-inflammatory cytokines, microglial and astrocyte reactivity, as well as the expression of proteins associated with BBB integrity (Figs. 3A and 6A). The results showed that in the periphery, HSB diet did not impairs the levels of IL-1β, IL-6, and TNF-α, but increased leptin levels, and INF-ɣ concentrations in the serum (Unpaired t-test test, IL-1β t_15_ = 0.9297, p = 0.3672; IL-6 t_16_ = 2.066, p = 0.0593; TNF-α t_10_ = 1.843, p = 0.0951; leptin t_9_ = 12.01, p < 0.0001; and INF-ɣ t_18_ = 2.334, p = 0.0314) (Fig. 2). Nonetheless, this fact was not observed in the hippocampus, where mRNA expression of inflammatory genes as well as protein levels of the cytokines evaluated remained unchanged in the HSB group (RT-PCR: Unpaired t-test test, IL-1β t_11_ = 0.007761, p = 0.9939; IL-6 t_12_ = 0.2917, p = 0.7755; TNF-α t_12_ = 0.8585, p = 0.4074; Mann–Whitney test, INF-ɣ, p = 0.2593; ELISA: IL-1β t_8_ = 0.0004175, p = 0.9997; IL-6 t_8_ = 0.4157, p = 0.6885; TNF-α t_8_ = 1.587, p = 0.1511; and INF-ɣ t_8_ = 1.985, p = 0.0824) (Fig. 3B–I). In accordance with these data, the comparison between Iba-1 optical densities, cell body to cell size ratio, as well as GFAP fluorescence intensity and percentage of GFAP stained area did not reveal significant differences between lean and obese groups (microglial optical density: unpaired t-test test, DG t_14_ = 0.1013, p = 0.9192; CA1 t_14_ = 0.1171, p = 0.9084; CA2–CA3 t_13_ = 0.6522, p = 0.5257; Microglial body to cell size ratio: Unpaired t-test test, DG t_14_ = 0.08705, p = 0.9319; Mann–Whitney test, CA1, p = 0.9329; CA2–CA3, p = 0.3282; GFAP fluorescence intensity: Mann–Whitney test, DG, p = 0.6991; unpaired t-test test, CA1 t_10_ = 0.3422, p = 0.7393; CA2–CA3 t_10_ = 0.7844, p = 0.4510; GFAP stained area: Mann–Whitney test, DG, p = 0.3095; unpaired t-test test, CA1 t_10_ = 0.3420, p = 0.7394; and CA2–CA3 t_10_ = 0.7843, p = 0.4510) (Figs. 4 and 5). Finally, HSB diet was not able to modulate the mRNA levels of Claudin-5, occludin, and ZO-1 levels, which suggest the regular maintenance of hippocampal BBB integrity in the obese mice group (unpaired t-test test, Claudin-5 t_17_ = 0.7456, p = 0.4661; Occludin t_17_ = 0.2230, p = 0.8262; and ZO-1 t_18_ = 0.9340, p = 0.3627) (Fig. 6B–D). Therefore, this dataset may suggest that the inflammatory features in our DIO model are present only in the periphery, and likely are not associated with the development of a hippocampal neuroinflammatory process.

### Chronic consumption of HSB diet promotes an increase in hippocampal glutamate release

**Figure 7:**
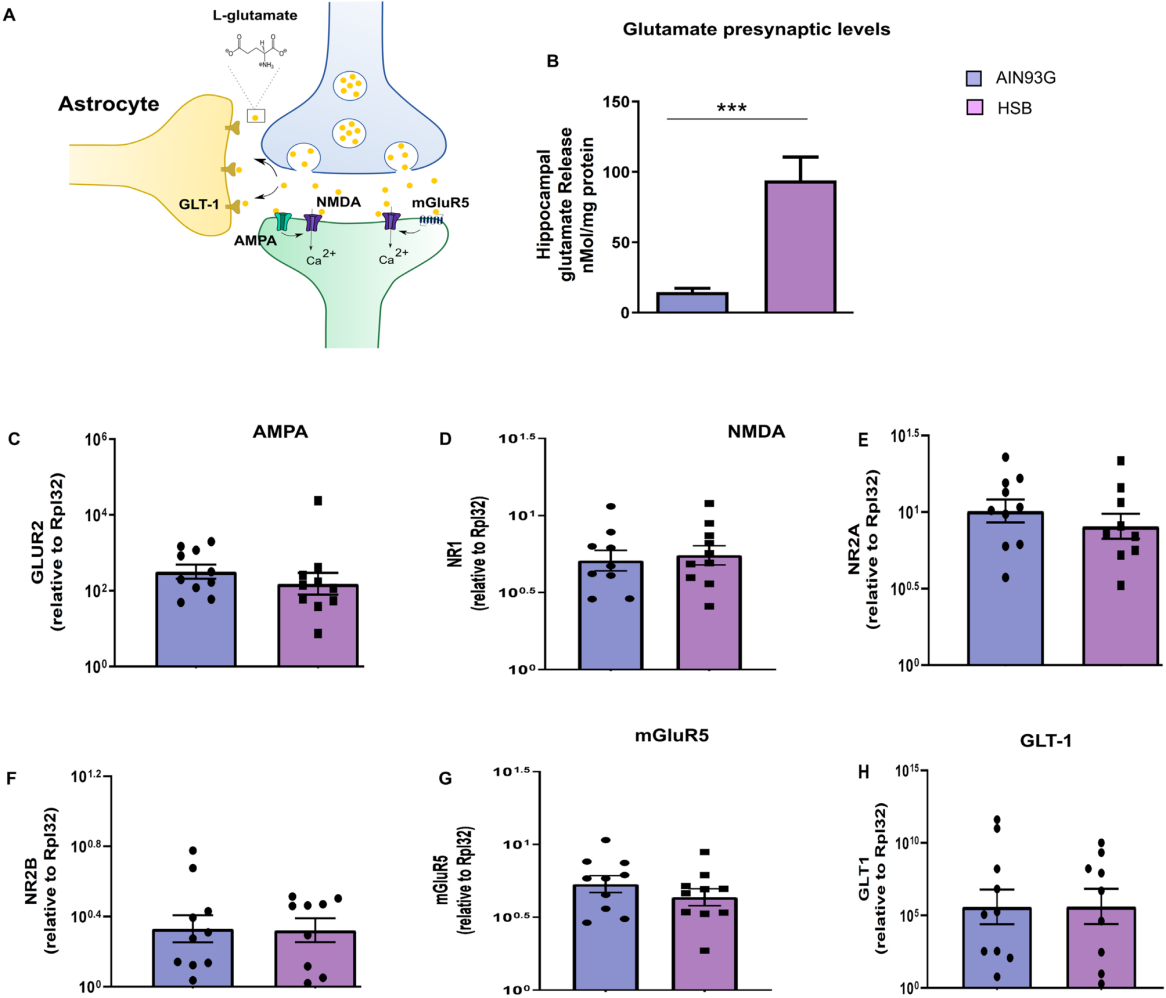
Chronic consumption of HSB diet increases hippocampal glutamate levels. **(A)** Schematic representation of tripartite glutamatergic neurotransmission. **(B)** Quantification of hippocampal synaptosomal glutamate levels. (**C**) mRNA levels for GLUR2 AMPA subunit. (**D–F**) mRNA levels for NR1, NR2A, and NR2B NMDA subunit. (**G**) mRNA levels for mGluR5. (**H**) Evaluation of mRNA levels from glial glutamate transporter (GLT-1). Error bars represent the mean ± SEM; n = 4 (**B**), and 9–10 (**C-H**). Unpaired two tail T-student test (**B–E,G,H)**, and Mann–Whitney test (**F)**. ***p < 0.001.

In order to verify the impact of the obesogenic environment on glutamatergic neurotransmission, we started our investigation by measuring hippocampal glutamate levels (Fig. 7). Notably, HSB diet increases hippocampal glutamate levels (Unpaired t-test test, hippocampal glutamate t_17_ = 4.003, p = 0.0009) (Fig. 7B**)**. Considering this, we decided to evaluate whether the increase of glutamate levels in the obese group might be associated with dysfunctional expression of AMPA and NMDA receptors by the quantification of functional subunits, respectively, GluR2, NR1, and NR2A/NR2B, as well the mRNA expression for mGluR5. Interestingly, our findings did not show remarkable differences between the diet groups (Unpaired t-test test, GluR2 t_18_ = 0.9338, p = 0.3627; NR1 t_17_ = 0.3773, p = 0.7106; NR2A t_17_ = 0.8998, p = 0.3808; mGluR5 t_18_ = 1.102, p = 0.2851; Mann–Whitney test, NR2B, p = 0.6305) (Fig. 7C–G). Finally, in order to evaluate whether excess of glutamate promoted by HSB diet was able to impair glutamatergic clearance in the synaptic cleft, we also quantify the mRNA levels of astrocyte glutamate transporter (GLT-1). Interestingly, we did not find alterations in the comparison between groups (Unpaired t-test test, GLT-1 t_17_ = 0.01760, p = 0.9862) (Fig. 7H).

### HSB diet up-regulates fractalkine but does not alter levels of neurotrophic factors and pre/post-synaptic plasticity proteins in the hippocampus

**Figure 8:**
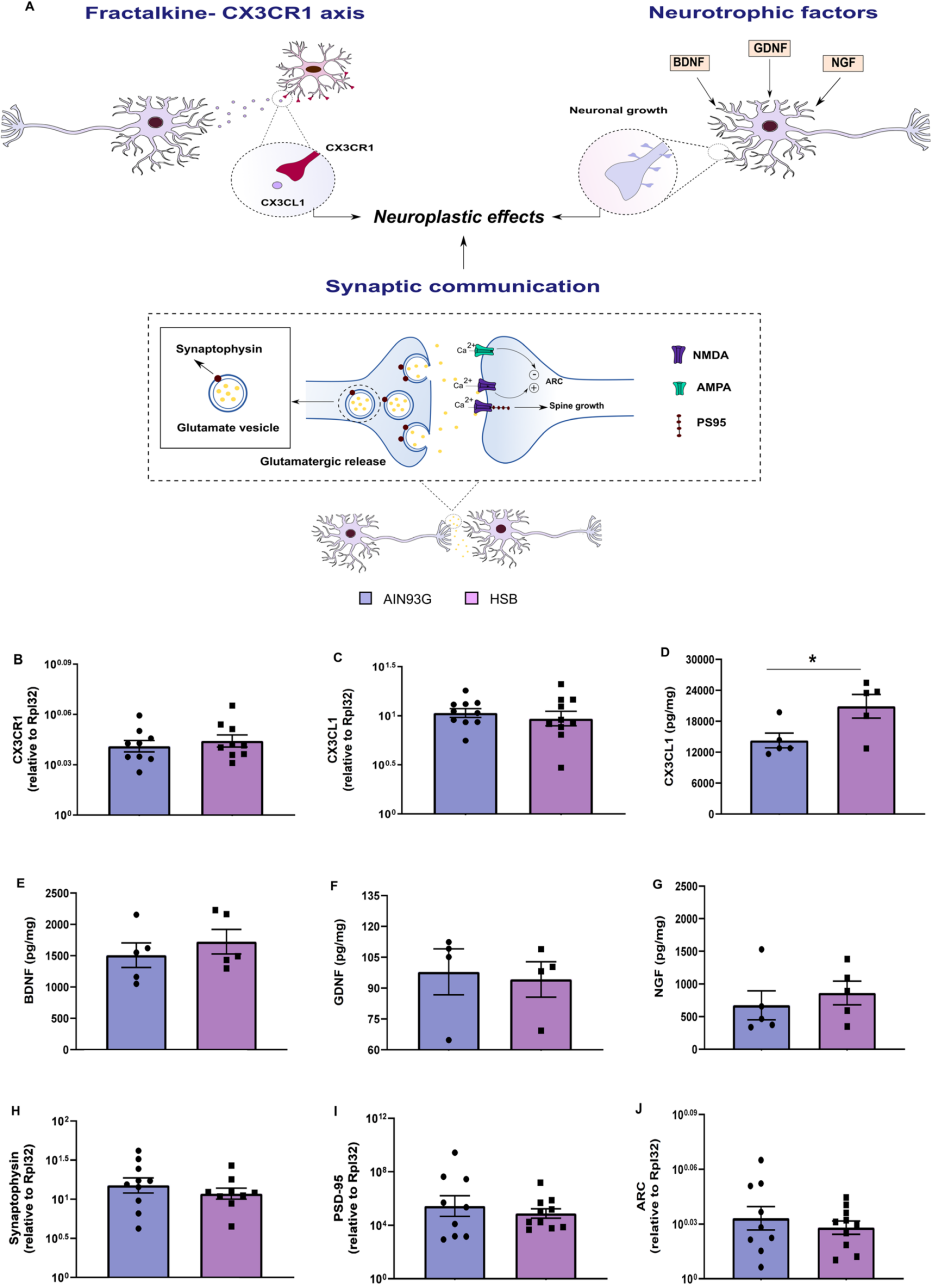
Chronic consumption of HSB diet increases hippocampal fractalkine levels. **(A)** Schematic representation of fractalkine neuron-glial communication and the control effect of neurotrophic factors, and pre/post synaptic proteins in neuronal synaptic plasticity. **(B,C)** Quantification of mRNA levels of fractalkine receptor (CX3CR1), and fractalkine (CX3CL1). (**D–G**) Measurements of protein levels of CX3CL1, brain derived neurotrophic factor (BDNF), nerve growth factor (NGF), and glial derived neurotrophic factor (GDNF). (**H–J**) Measurements of mRNA levels of synaptophysin, postsynaptic density protein 95 (PSD-95), and activity-regulated cytoskeleton-associated protein (ARC). Error bars represent the mean ± SEM; n = 4 (**F**), 5 (**D,E,G**), and 9–10 (**B,C,H–J**). Unpaired two tail T-student test (**B–E,H–J)**, and Mann–Whitney test (**F–G**). *p < 0.05.

The Fractalkine/CX3CR1 axis is associated with microglia and neuronal crosstalk by regulating synaptic plasticity and glutamatergic neurotransmission, which provides implications in several pathological conditions of memory^46^. In addition, neurotrophic factors, pre/post synaptic proteins, as well as glutamate levels interact with each other to regulate neuroplasticity, providing a key role in memory regulation^26,47^. Thus, we decided to conduct a preliminary investigation, a posteriori, to elucidate whether the obesogenic environment associated with HSB diet might cause alterations in the Fractalkine/CX3CR1 axis, besides in the levels of neurotrophic factors, and pre/post synaptic proteins associated with memory regulation and plasticity (Fig. 8A). For this, we measured hippocampal levels of fractalkine, CX3CR1, BDNF, NGF, GDNF, synaptophysin, PDS-95 and ARC (Fig. 8B–J). Our results showed that mRNA levels of fractalkine and CX3CR1 were unaffected by HSB diet (Unpaired t-test test, Fractalkine t_18_ = 0.6510, p = 0.5233; Mann–Whitney test, CX3CR1, p = 0.6842) (Fig. 8B, C). However, we observed that hippocampal fractalkine protein levels were up-regulated in obese mice (unpaired t-test, t_16_ = 2.451, p = 0.0399) (Fig. 8D). Regarding neurotrophic factors, synaptophysin, PSD-95, and ARC, our preliminary data reveal no changes in any of the markers assessed (ELISA: unpaired t-test test, BDNF t_8_ = 0.7803, p = 0.4577; Mann–Whitney test, NGF, p = 0.5476; and GDNF, p = 0.4857; RT-PCR: unpaired t-test test, Synaptophysin t_17_ = 0.8716, p = 0.3956; PSD-95 t_8_ = 0.6794, p = 0.5060; ARC t_17_ = 0.7103, p = 0.4871) (Fig. 8E–J).

## Discussion

**Figure 9:**
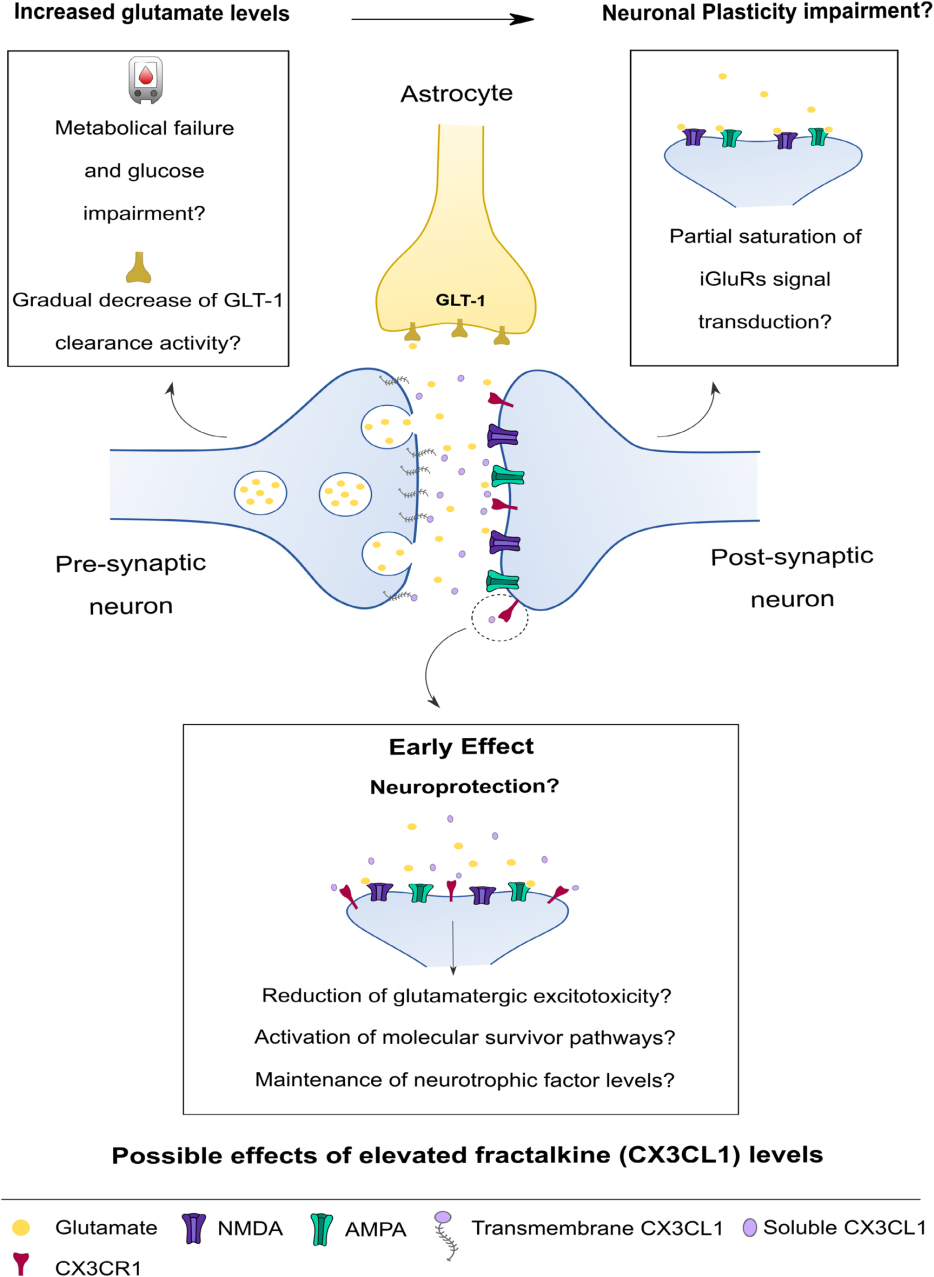
Possible mechanism associated with hippocampal glutamatergic neurotransmission dysfunction triggered by diet-induced obesity. We hypothesized that an earlier GLT-1 compensatory mechanism, associated with glucose metabolic failure, increases cortical and hippocampal glutamatergic levels. In this context, we believe that the persistence of metabolic abnormalities may impact GLT-1 clearance efficiency, leading to an increase in glutamate levels and, saturation of postsynaptic glutamatergic receptor activity, which may lead to an impairment of synaptic plasticity, and dysfunction in the plastic capacity of the hippocampal network. Thus, we hypothesized that these mechanisms may increase the levels of soluble fractalkine, in order to limit a possible hippocampal synaptic damage promoted by glutamatergic toxicity.

The main idea of this work was to understand how an obesogenic inflammatory environment associated with prolonged consumption of a highly palatable/ energy-dense diet, may impact central neurotransmission and its functionality. For that, we have demonstrated that chronic exposure to HSB diet promotes hippocampal-dependent memory impairment and increases glutamate levels in the hippocampus of obese mice. Although we observed elevated levels of peripheral inflammatory markers typical of obesity, such as leptin, and INF-ɣ, in the hippocampus we did not observe damage to the integrity of the BBB or neuroinflammatory features associated with exposure to an obesogenic diet, which overturned our hypothesis that increased glutamate levels in the hippocampus could be associated with hippocampal neuroinflammatory mechanisms triggered by obesogenic peripheral inflammation (Figs. 2, 3, 4, 5 and 6). In this milieu, highly palatable/ energy-dense foods are strongly associated with glucose metabolism impairment and diabetes mellitus type 2 (TDM2)^11,48^. An interesting study demonstrated that the co-association between obesity and TDM2 was able to reduce the molecular carbon labeling of glutamate in the hippocampus and cortex, which could be associated with decrease in glucose metabolism in the tricarboxylic acid (TCA) cycle, generating biochemical dysfunctions in glutamate production^48^. In accordance with that, exposure to a high-fat or high fat-sucrose diet may decrease glutamine synthase activity, glutamate/glutamine levels, and up-regulate GLT-1 expression and activity, which may suggest that these dietary components by themselves, could impact glutamate metabolism^33,34^. Considering that glutamatergic recycling is a process that requires a large energy input, it is possible that the increase of GLT-1 expression and/or activity acts as a compensatory mechanism in order to improve the glutamatergic recycling efficiency and also vesicular glutamate levels in a metabolic failure scenario^33,34,49^. However, it is important to note that it has been suggested that the GLT-1 clearance efficiency tends to decrease gradually after persistent metabolic dysfunctions or increased neuroinflammation, which may predispose to glutamatergic excitotoxicity and memory decline^32,49^. Therefore, considering that in similar DIO studies with a shorter diet exposure time, as well as the fact that the HSB diet induces a metabolic impairment without affecting GLT-1 expression (Fig. 7H and Fig. S2), we hypothesized that the increase in hippocampal glutamate levels may be a result of an earlier GLT-1 compensatory mechanism due to the persistence of metabolic failure (Fig. 9).

It has been reported that increased glutamate levels are associated with impairment in synaptic plasticity, including abnormal formation of LTP and long-term depression (LTD), in a mechanism involving overactivation of ionotropic and metabotropic receptors, increased Ca^+2^ signaling, decreased production of neurotrophic factors, synaptic proteins, among others^25,26,50^. Interestingly, in DIO models, LTP and LTD phenomena are decreased in CA1-CA3 region, which may be associated with acquisition and reconsolidation dysfunctions of aversive and episodic memory, suggesting that an obesogenic environment may increase the vulnerability to a poor synaptic plasticity regulation and cognitive impairment^45,51^. Although none of these studies evaluated whether decrease in synaptic plasticity and memory were associated with glutamatergic neurotransmission, it has been demonstrated that HFD promotes a downregulation of NMDA hippocampal NR2B subunit, which may lead to dysfunctions in synaptic plasticity dependent on LTD, likely contributing to plastic decline regulation in an obesity environment^34^. Here, we did not observe changes in the levels of expression of NMDA and AMPA subunits, as well as other classical receptors that also may contribute to the regulation of LTP and LTD, such as mGluR5 (Fig. 7C–G). However, we cannot rule out the possibility that the functional pattern of these receptors may be altered. In this context, it is well known that the saturation of iGluR activity, due to high glutamate levels, can disrupt the formation of new LTP and LTD patterns, contributing to the weakening of reinforcing traces of memories, new memory encoding, and learning^52,53^. Interestingly, our behavior dataset suggest that the main deleterious effects of the HSB diet is concentrated on processes associated with the reinforcement of consolidated memories, in the case of ORT and OLT, and new learning (memory extinction) (Fig. 1D–E, H, I). Thus, although we did not assess the hippocampal pattern of LTP/LTD, which is an important limitation of our work, we hypothesized that increased glutamate levels might induce the saturation of glutamatergic receptor activity, promoting an impairment of synaptic plasticity, as well as limiting the plastic capacity of the hippocampal network, which could impair signal transduction associated with refinement and extinction of an acquired memory. Nevertheless, the absence of behavioral changes observed in the Y maze test, aversive memory evocation, and social recognition suggests that possibly primary memory processes such as acquisition, consolidation, and retrieval could still be preserved (Fig. 1B, G, and Fig. S4). Although speculative, we believe that two alternatives might support this phenomenon: (I) Impairment of synaptic plasticity, promoted by the elevation of glutamate levels is not strong enough to saturate the signal transduction associated with acquisition and consolidation. However, increased glutamate levels can limit consolidated circuits and the full acquisition of new information relative to a familiar context. (II) Compensatory systems may be attempting to partially protect hippocampal circuitry from the potentially deleterious effects of increased glutamate levels on plasticity (Fig. 9).

Fractalkine, a chemokine abundantly produced by neurons, belonging to the CX3C chemokine family, acts in several synaptic plastic processes and also contributes to memory and learning in physiological conditions^54,55^. Furthermore, the effects of fractalkine-CX3CR1 signaling have been pointed out as a potential neuroprotective path in conditions associated with neurotoxicity and memory decline, such as Alzheimer´s and Parkinson´s disease^56,57^. In this sense, in vitro experiments showed that increased hippocampal fractalkine levels are associated with neuroprotective effects against glutamatergic excitotoxicity in a mechanism that involves the reduction of postsynaptic AMPA-evoked currents, increased expression of synaptic NR2A/NMDA, activation of survivor pathways, and production of neurotrophic factors, such as BDNF^58–60^. Moreover, it was demonstrated that CX3CR1 expression in hippocampal neurons mediated the neurotrophic effects of fractalkine in a viral neurotoxicity model^61^. Interestingly, in a murine model for Alzheimer's disease, it was reported that the overexpression of fractalkine reversed the decline in hippocampal synaptophysin^62^. Furthermore, lower levels of fractalkine-CX3CR1 signaling in the hippocampus of obese or lean mice treated with a CX3CR1 antagonist are associated with decreased levels of PSD-95 and cognitive deficits^18^. Importantly, we observed that HSB diet exposition can increase hippocampal fractalkine levels, but did not change the concentration of neurotrophic factors, as well as the mRNA expression of synaptophysin, PSD-95, and ARC (Fig. 8). Perhaps this fact is due to neuroprotective fractalkine effects in response to increased glutamate levels, which could be evidence in favor of the compensatory neuroprotective hypothesis previously raised. It is also important to point out that although we observed an increase in the protein levels of fractalkine, the expression of fractalkine mRNA remains unchanged in HSB group (Fig. 8). This is in accordance with in vitro reports, where glutamate-induced neurotoxicity did not affect fractalkine gene expression but increases soluble fractalkine levels, which may indicate that the neurotoxic environment may promote fractalkine cleavage from the neuronal membrane^63^. Interestingly, although fractalkine can signal as a membrane-bound protein or a soluble fragment, some studies suggest that the soluble form is more associated with neuroprotective effects^57,64^. Therefore, we hypothesize that the increased levels of fractalkine may be an attempt to limit a possible hippocampal synaptic damage promoted by glutamate (Fig. 9). However, further experiments including the molecular investigation of survival and cell growth pathways modulated by fractalkine may help to support this hypothesis.

It is critical to underscore the limitations of our work. Firstly, due to our focus on a single time point, our current dataset lacks the ability to establish definitive conclusions about the connection between glutamatergic dysfunction and neuroinflammation. While in this study we observed higher glutamate levels in the hippocampus, this elevation might be the outcome of a process not directly linked to neuroinflammation but rather related to metabolic dysfunction, as hypothesized earlier. However, due to the limitations of our experimental design, we cannot ascertain whether the absence of neuroinflammation was a result of a previous neuroinflammatory process or a subsequent response to increased glutamate levels in the hippocampus. It remains uncertain whether a potential neuroinflammatory process could be initiated independently of peripheral inflammation. Taking this into consideration, it is important to point out that the chronology of neuroinflammation in obesity is still poorly known, and that the initiation of this phenomenon appears to be related with a non-linear temporal dynamics based on a variety of circumstances, including the ingredients and centesimal composition of the diets, duration of diet exposure, as well as the age of onset of dietary exposure^65–68^. In this milieu, a study pointed out that a short-term exposure to a HFD 60%, from 2 to 7 days, was associated with a decrease in synaptophysin expression, increase in TNF-α, IL-6 levels, astrocyte reactivity and BBB permeability in the hippocampus, suggesting that this region may be quickly affected by damage induced by highly palatable and energy-dense foods^69^. Furthermore, emerging evidence suggests that post-weaning exposure to a HFD containing 60% of calories from fat, 20% from protein, and 20% from carbohydrates for duration of 6 weeks can induce neuroinflammatory features. Additionally, this HFD regimen has been associated with reduced levels of GLUA1 and GLUA2, which are important subunits of the AMPA receptors^67^. Moreover, the enduring impact of alterations in this setting may have long-term consequences, as it can lead to sustained high levels of glutamate, decrease the expression of vesicular glutamate transporter (vGLUT), trigger ongoing astrocyte reactivity in the hippocampus, and result in memory impairment^70^. Interestingly, in an intriguing study conducted over a span of eight weeks, researchers implemented a diet closely resembling to ours. Notably, the results revealed no significant alterations in dendritic spine density, neuroinflammation, or glutamate dysfunction within the hippocampus. Nevertheless, the authors reported that this HFD was able to induce cognitive decline along with all the aforementioned molecular dysfunctions in the prefrontal cortex^6^. Thus, while our findings are not conclusive, it is tempting to suggest that prolonged exposure to HSB diet might cause acute neuroinflammation in the hippocampus which may predispose to late changes in glutamatergic signaling. Nonetheless, the absence of studies examining the kinetics of hippocampal neuroinflammation associated with an obesogenic environment leaves us with insufficient concrete evidence to speculate on this possibility. Furthermore, it is essential to consider that several other brain structures implicated in memory processes, including the cortex, cerebellum, and amygdala, can also experience neuroinflammatory states as a result of both short-term and long-term exposure to a poor diet^6,42,65^. Also, we must recognize that other brain regions might indeed exhibit neuroinflammatory and glutamatergic signaling changes consistent with our initial hypothesis. However, it is important to emphasize that such possibilities extend beyond the limits supported by the data we have gathered in our study. Other important limitations of our study are the facts that we did not demonstrate whether the pattern activity of iGluR was affected by the obesogenic environment, and whether our DIO model presented damages in potential molecular pathways linked to neurotoxicity, which makes it difficult to understand potential changes associated with plastic mechanisms. Finally, we did not measure parameters such as sex-specific differences as well as molecular pathways associated with the neuroprotective effect of fractalkine. Thus, a deeper investigation of the plastic electrophysiological parameters, pathways associated to glutamate-fractalkine signaling, neuroinflammatory kinetics, and sex differences, is needed in order to elucidate the nature of these highlights.

### Supplementary Information

Supplementary Information.

## Data Availability

The datasets generated during and/or analyzed during the current study are available from the corresponding author on reasonable request. Drawings: All drawings were manually performed using the inkscape software (https://inkscape.org/about/overview/).

## References

[1] Askari M, Heshmati J, Shahinfar H, Tripathi N, Daneshzad E (2020). Ultra-processed food and the risk of overweight and obesity: A systematic review and meta-analysis of observational studies. Int. J. Obes..

[2] Cheke LG, Bonnici HM, Clayton NS, Simons JS (2017). Obesity and insulin resistance are associated with reduced activity in core memory regions of the brain. Neuropsychologia.

[3] Prickett C, Brennan L, Stolwyk R (2015). Examining the relationship between obesity and cognitive function: A systematic literature review. Obes. Res. Clin. Pract..

[4] Takase K, Tsuneoka Y, Oda S, Kuroda M, Funato H (2016). High-fat diet feeding alters olfactory-, social-, and reward-related behaviors of mice independent of obesity. Obesity.

[5] Melo HM (2020). Palmitate is increased in the cerebrospinal fluid of humans with obesity and induces memory impairment in mice via pro-inflammatory TNF-α. Cell Rep..

[6] Bocarsly ME (2015). Obesity diminishes synaptic markers, alters microglial morphology, and impairs cognitive function. PNAS.

[7] Forte N (2021). Orexin-A and endocannabinoids are involved in obesity-associated alteration of hippocampal neurogenesis, plasticity, and episodic memory in mice. Nat. Commun..

[8] Reichelt AC, Maniam J, Westbrook RF, Morris MJ (2015). Dietary-induced obesity disrupts trace fear conditioning and decreases hippocampal reelin expression. Brain Behav. Immun..

[9] Butler MJ (2021). The role of Western diets and obesity in peripheral immune cell recruitment and inflammation in the central nervous system. Brain, Behav. Immun.-Health.

[10] Ransohoff RM, Schafer D, Vincent A, Blachère NE, Bar-Or A (2015). Neuroinflammation: Ways in which the immune system affects the brain. Neurotherapeutics.

[11] Maioli TU (2016). High sugar and butter (HSB) diet induces obesity and metabolic syndrome with decrease in regulatory T cells in adipose tissue of mice. Inflamm. Res..

[12] Miller AA, Spencer SJ (2014). Obesity and neuroinflammation: A pathway to cognitive impairment. Brain Behav. Immun..

[13] Ogata S, Ito S, Masuda T, Ohtsuki S (2019). Changes of blood-brain barrier and brain parenchymal protein expression levels of mice under different insulin-resistance conditions induced by high-fat diet. Pharm. Res..

[14] Alexaki VI (2021). The impact of obesity on microglial function: Immune, metabolic and endocrine perspectives. Cells.

[15] Liddelow SA (2017). Neurotoxic reactive astrocytes are induced by activated microglia. Nature.

[16] Cope EC (2018). Microglia play an active role in obesity-associated cognitive decline. J. Neurosci..

[17] Hao S, Dey A, Yu X, Stranahan AM (2016). Dietary obesity reversibly induces synaptic stripping by microglia and impairs hippocampal plasticity. Brain Behav. Immun..

[18] Kawamura N (2021). Impaired brain fractalkine-CX3CR1 signaling is implicated in cognitive dysfunction in diet-induced obese mice. BMJ Open Diabetes Res. Care.

[19] Sobesky JL (2014). High-fat diet consumption disrupts memory and primes elevations in hippocampal IL-1β, an effect that can be prevented with dietary reversal or IL-1 receptor antagonism. Brain Behav. Immun..

[20] Debiec J, LeDoux JE, Nader K (2002). Cellular and systems reconsolidation in the hippocampus. Neuron.

[21] Quirk GJ, Mueller D (2008). Neural mechanisms of extinction learning and retrieval. Neuropsychopharmacology.

[22] Zyuzina AB, Balaban PM (2017). Extinction and reconsolidation of memory. Neurosci. Behav. Physiol..

[23] Abel T, Lattal KM (2001). Molecular mechanisms of memory acquisition, consolidation and retrieval. Curr. Opin. Neurobiol..

[24] Riedel G, Platt B, Micheau J (2003). Glutamate receptor function in learning and memory. Behav. Brain Res..

[25] Katagiri H, Tanaka K, Manabe T (2001). Requirement of appropriate glutamate concentrations in the synaptic cleft for hippocampal LTP induction. Eur. J. Neurosci..

[26] Mattson MP (2008). Glutamate and neurotrophic factors in neuronal plasticity and disease. Ann. N. Y. Acad. Sci..

[27] Barger SW, Goodwin ME, Porter MM, Beggs ML (2007). Glutamate release from activated microglia requires the oxidative burst and lipid peroxidation. J. Neurochem..

[28] Mizuno T (2008). Interferon-γ directly induces neurotoxicity through a neuron specific, calcium-permeable complex of IFN-γ receptor and AMPA GluRl receptor. FASEB J..

[29] Viviani B (2003). Interleukin-1β enhances NMDA receptor-mediated intracellular calcium increase through activation of the Src family of kinases. J. Neurosci..

[30] Stellwagen D, Beattie EC, Seo JY, Malenka RC (2005). Differential regulation of AMPA receptor and GABA receptor trafficking by tumor necrosis factor-α. J. Neurosci..

[31] Haroon E, Miller AH, Sanacora G (2017). Inflammation, glutamate, and glia: A trio of trouble in mood disorders. Neuropsychopharmacology.

[32] Tsai SF (2018). High-fat diet suppresses the astrocytic process arborization and downregulates the glial glutamate transporters in the hippocampus of mice. Brain Res..

[33] Martínez-Orozco H (2021). High-fat and combined high-fat–high-fructose diets impair episodic-like memory and decrease glutamate and glutamine in the hippocampus of adult mice. Nutr. Neurosci..

[34] Valladolid-Acebes I (2012). High-fat diets induce changes in hippocampal glutamate metabolism and neurotransmission. Am. J. Physiol.-Endocrinol. Metab..

[35] Nascimento AF (2008). A hypercaloric pellet-diet cycle induces obesity and co-morbidities in wistar rats. Arq. Bras. Endocrinol. Metabol..

[36] Denninger JK, Smith BM, Kirby ED (2019). Novel object recognition and object location behavioral testing in mice on a budget. J. Vis. Exp..

[37] Amaral-Júnior PA (2019). A custom microcontrolled and wireless-operated chamber for auditory fear conditioning. Front. Neurosci..

[38] Almeida-Santos AF (2019). Social isolation impairs the persistence of social recognition memory by disturbing the glutamatergic tonus and the olfactory bulb-dorsal hippocampus coupling. Sci. Rep..

[39] Pedro, P. F., Tsakmaki, A. & Bewick, G. A. *The Glucose Tolerance Test in Mice*. *Animal Models of Diabetes: Methods and Protocols, Methods in Molecular Biology*. Vol. 2128 (2020). 10.1007/978-1-0716-0385-7_14 32180195

[40] Virtue S, Vidal-Puig A (2021). GTTs and ITTs in mice: Simple tests, complex answers. Nat. Metab..

[41] Hovens I, Nyakas C, Schoemaker R (2014). A novel method for evaluating microglial activation using ionized calcium-binding adaptor protein-1 staining: Cell body to cell size ratio. Neuroimmunol. Neuroinflamm..

[42] Gomes JAS (2020). High-refined carbohydrate diet consumption induces neuroinflammation and anxiety-like behavior in mice. J. Nutr. Biochem..

[43] Dunkley PR, Jarvie PE, Heath JW, Kidd GJ, Rostas JAP (1986). A rapid method for isolation of synaptosomes on Percoll gradients. Brain Res..

[44] Nicholls DG, Sihra TS, Sanchez-Prieto J (1987). Calcium-dependent and-independent release of glutamate from synaptosomes monitored by continuous fluorometry. J. Neurochem..

[45] Hwang LL (2010). Sex differences in high-fat diet-induced obesity, metabolic alterations and learning, and synaptic plasticity deficits in mice. Obesity.

[46] Luo P, Chu SF, Zhang Z, Xia CY, Chen NH (2019). Fractalkine/CX3CR1 is involved in the cross-talk between neuron and glia in neurological diseases. Brain Res. Bull..

[47] Iasevoli F, Tomasetti C, De Bartolomeis A (2013). Scaffolding proteins of the post-synaptic density contribute to synaptic plasticity by regulating receptor localization and distribution: Relevance for neuropsychiatric diseases. Neurochem. Res..

[48] Sickmann HM, Waagepetersen HS, Schousboe A, Benie AJ, Bouman SD (2010). Obesity and type 2 diabetes in rats are associated with altered brain glycogen and amino-acid homeostasis. J. Cereb. Blood Flow Metab..

[49] Andersen JV (2021). Glutamate metabolism and recycling at the excitatory synapse in health and neurodegeneration. Neuropharmacology.

[50] Barnes JR (2020). The relationship between glutamate dynamics and activity-dependent synaptic plasticity. J. Neurosci..

[51] Spinelli M (2017). Brain insulin resistance impairs hippocampal synaptic plasticity and memory by increasing GluA1 palmitoylation through. Nat. Commun..

[52] Martin SJ, Grimwood PD, Morris RGM (2000). Synaptic plasticity and memory: An evaluation of the hypothesis. Annu. Rev. Neurosci..

[53] Moser EI, Moser MB (1999). Is learning blocked by saturation of synaptic weights in the hippocampus?. Neurosci. Biobehav. Rev..

[54] Lauro C, Catalano M, Trettel F, Limatola C (2015). Fractalkine in the nervous system: Neuroprotective or neurotoxic molecule?. Ann. N. Y. Acad. Sci..

[55] Rogers JT (2011). CX3CR1 deficiency leads to impairment of hippocampal cognitive function and synaptic plasticity. J. Neurosci..

[56] Chen P, Zhao W, Guo Y, Xu J, Yin M (2016). CX3CL1/CX3CR1 in Alzheimer’s disease: A target for neuroprotection. Biomed. Res. Int..

[57] Morganti JM (2012). The soluble isoform of CX3CL1 is necessary for neuroprotection in a mouse model of Parkinson’s disease. J. Neurosci..

[58] Lauro C (2015). Fractalkine/CX3CL1 engages different neuroprotective responses upon selective glutamate receptor overactivation. Front. Cell. Neurosci..

[59] Ragozzino D (2006). Chemokine fractalkine/CX3CL1 negatively modulates active glutamatergic synapses in rat hippocampal neurons. J. Neurosci..

[60] Limatola C (2005). Chemokine CX3CL1 protects rat hippocampal neurons against glutamate-mediated excitotoxicity. J. Neuroimmunol..

[61] Meucci O, Fatatis A, Simen AA, Miller RJ (2000). Expression of CX3CR1 chemokine receptors on neurons and their role in neuronal survival. PNAS.

[62] Fan Q (2020). Activated CX3CL1/Smad2 signals prevent neuronal loss and Alzheimer’s tau pathology-mediated cognitive dysfunction. J. Neurosci..

[63] Chapman GA (2000). Fractalkine cleavage from neuronal membranes represents an acute event in the inflammatory response to excitotoxic brain damage. J. Neurosci..

[64] Winter AN (2020). Two forms of CX3CL1 display differential activity and rescue cognitive deficits in CX3CL1 knockout mice. J. Neuroinflamm..

[65] Guillemot-Legris O (2016). High-fat diet feeding differentially affects the development of inflammation in the central nervous system. J. Neuroinflamm..

[66] Guillemot-Legris O, Muccioli GG (2017). Obesity-induced neuroinflammation: Beyond the hypothalamus. Trends Neurosci..

[67] Yang Y (2019). Early-life high-fat diet-induced obesity programs hippocampal development and cognitive functions via regulation of gut commensal *Akkermansia muciniphila*. Neuropsychopharmacology.

[68] Beilharz JE, Kaakoush NO, Maniam J, Morris MJ (2016). The effect of short-term exposure to energy-matched diets enriched in fat or sugar on memory, gut microbiota and markers of brain inflammation and plasticity. Brain Behav. Immun..

[69] de Paula GC (2021). Hippocampal function is impaired by a short-term high-fat diet in mice: Increased blood–brain barrier permeability and neuroinflammation as triggering events. Front. Neurosci..

[70] Hascup ER (2019). Diet-induced insulin resistance elevates hippocampal glutamate as well as VGLUT1 and GFAP expression in AβPP/PS1 mice. J. Neurochem..

