# Supplementary material for: Chronic hyperpalatable diet induces impairment of hippocampal-dependent memories and alters glutamatergic and fractalkine axis signaling
Source: Sci Rep. 2023 Sep 29;13:16358. doi: 10.1038/s41598-023-42955-9 (PMC10541447; doi:10.1038/s41598-023-42955-9)
Supplement: Supplementary file 1 — Supplementary Information. [file 41598_2023_42955_MOESM1_ESM.doc]

**CHRONIC HYPERPALATABLE DIET INDUCES IMPAIRMENT OF HIPPOCAMPAL-DEPENDENT MEMORIES** **AND ALTERS GLUTAMATERGIC AND FRACTALKINE AXIS SIGNALING.**

Roberta Ribeiro^a^, Emanuele Guimarães Silva^c^, Felipe Caixeta Moreira^c^, Giovanni Freitas Gomes^f^, Gabriela Reis Cussat^a^, Barbara Stehling Ramos Silva^a^, Maria Carolina Machado da Silva^a^, Heliana de Barros Fernandes^d^, Carolina de Sena Oliveira^a^, Leonardo de Oliveira Guarnieri^b^, Victoria Lopes^e^, Cláudia Natália Ferreira^e^, Ana Maria Caetano de Faria^c^, Tatiani Uceli Maioli^c^, Fabíola Mara Ribeiro^c^, Aline Silva de Miranda^d^, Grace Schenatto Pereira Moraes^b^, Antônio Carlos Pinheiro de Oliveira^a^ e Luciene Bruno Vieira^a^

^a^ Department of Pharmacology, ICB, Federal University of Minas Gerais, Belo Horizonte, Brazil

^b^ Department of Physiology and Biophysics, ICB, University of Minas Gerais, Belo Horizonte, Brazil

^c^ Department of Immunology and Biochemistry, ICB, University of Minas Gerais, Belo Horizonte, Brazil

^d^ Department of Morphology, ICB, University of Minas Gerais, Belo Horizonte, Brazil

^e^ Colégio Técnico, University of Minas Gerais, Belo Horizonte, Brazil

^f^ Center of Research in Inflammatory Diseases, Ribeirão Preto Medical School, University of São Paulo, Ribeirão Preto, Brazil

**SUPPLEMENTARY DATA**

**
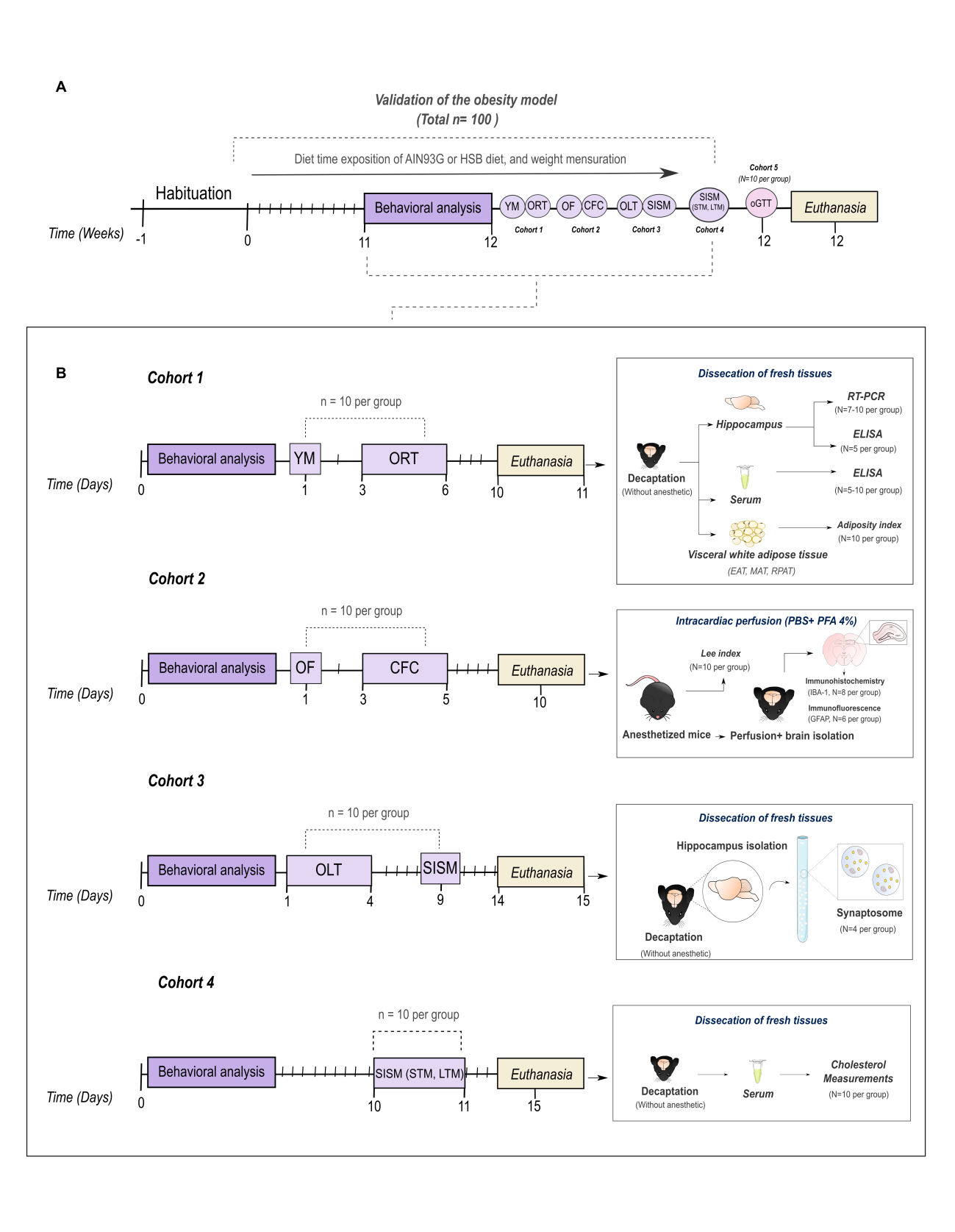
**

**Figure S1 -** **Schematic representation of experimental design.** **(A)** General scheme of experimental design. A total of 100 animals were exposed to AIN93G or HSB diet for 12 weeks. Five different mice cohorts were utilized for experimental procedures which included 4 mice cohorts for behavioral experiments, and one cohort for oral glucose tolerance test (**oGTT**). (**B**) Description of behavioral cohort experimental procedures, euthanasia and molecular analysis. **Abbreviations: YM:** Y maze test; **ORT:** Object Recognition Test; **OF:** Open Field test; **CFC:** Contextual fear conditioning; **OLT:** Object Location Test; **SISM:** Social interaction and social memory test (Three chamber paradigm); **SISM (STM, LTM):** interaction and social memory test (one chamber paradigm), **STM:** Short term-memory, **LTM:** Long term memory.

**
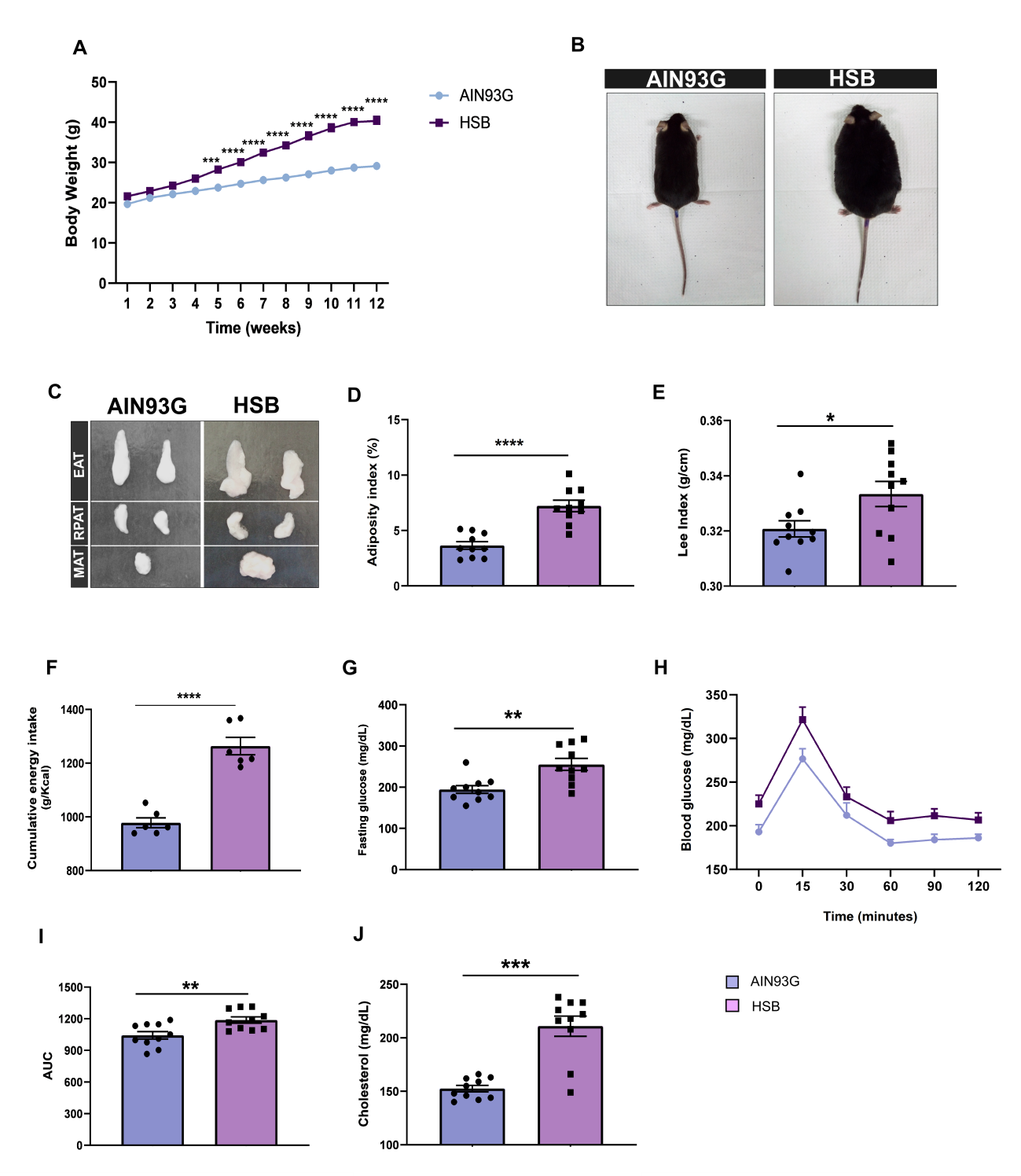
Figure S2. Chronic consumption of HSB diet induces an increase in body weight and metabolic disturbances in C57BL/6J mice.** (**A**) Body weight measurements of AIN93G and HSB groups showed a significant time x diet interaction effect (F_(11, 456)_ = 15,42). (**B-C**) The representative phenotype of interest groups and isolated visceral adipose tissues (epididymal (**EAT**), mesenteric (**MAT**) and retroperitoneal (**RPAT**)) at 12^th^ week of the protocol. (**D-E**) Measurements of the adiposity (%) and Lee index (g/cm). (**F**) Cumulative energy intake (g/Kcal) per cage. (**G**) Evaluation of the glucose levels (mg/dL) after 6 h of fasting. (**H-I**) Representative oGTT curve and evaluation of the Area under curve (AUC) of blood glucose. (**J**) Total cholesterol levels. Error bars represent the mean ± SEM; n = 40 (**Figure A**), n=10 (**Figures D,E,G,H,I,J**), and n=6 (**figure F**). Two-way ANOVA followed by Tukey’s post-test (**A**), Unpaired two tail T-student test (**D**,**E**,**F**,**G,I**), Mann Whitney test (**J**). *p <0.05; **p <0.01; ***p <0.001; ****p <0.0001.

**
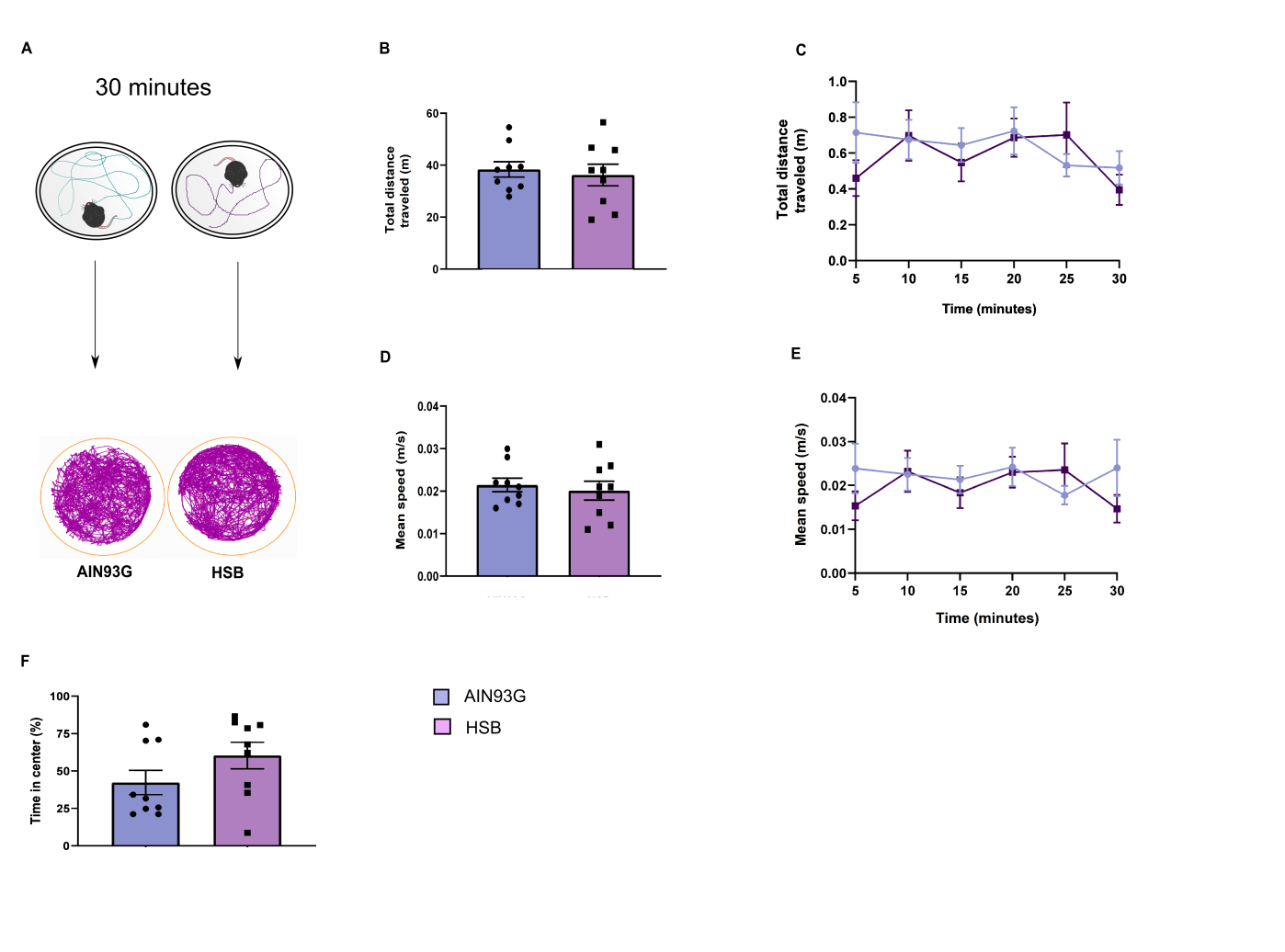
**

**Figure S3. Chronic consumption of HSB does not alter locomotor and exploratory behaviors. (A-F)** Evaluation of locomotor and exploratory behavior in Open field test: **(A)** Schematic representation of method used in the open field test and representative track plot of exploratory behavior in all behavioral test time (30 minutes). **(B-C)** Computation and representative curve of total traveled distance (m), respectively. **(D-E)** Computation, and representative curve of the mean of speed (m/s) of the interesting groups. **(F)** Evaluation of the percentage time at the center of open field apparatus. Error bars represent the mean ± SEM; n =9-10. Unpaired two tail T-student test (**B, D,** and **F**).

**
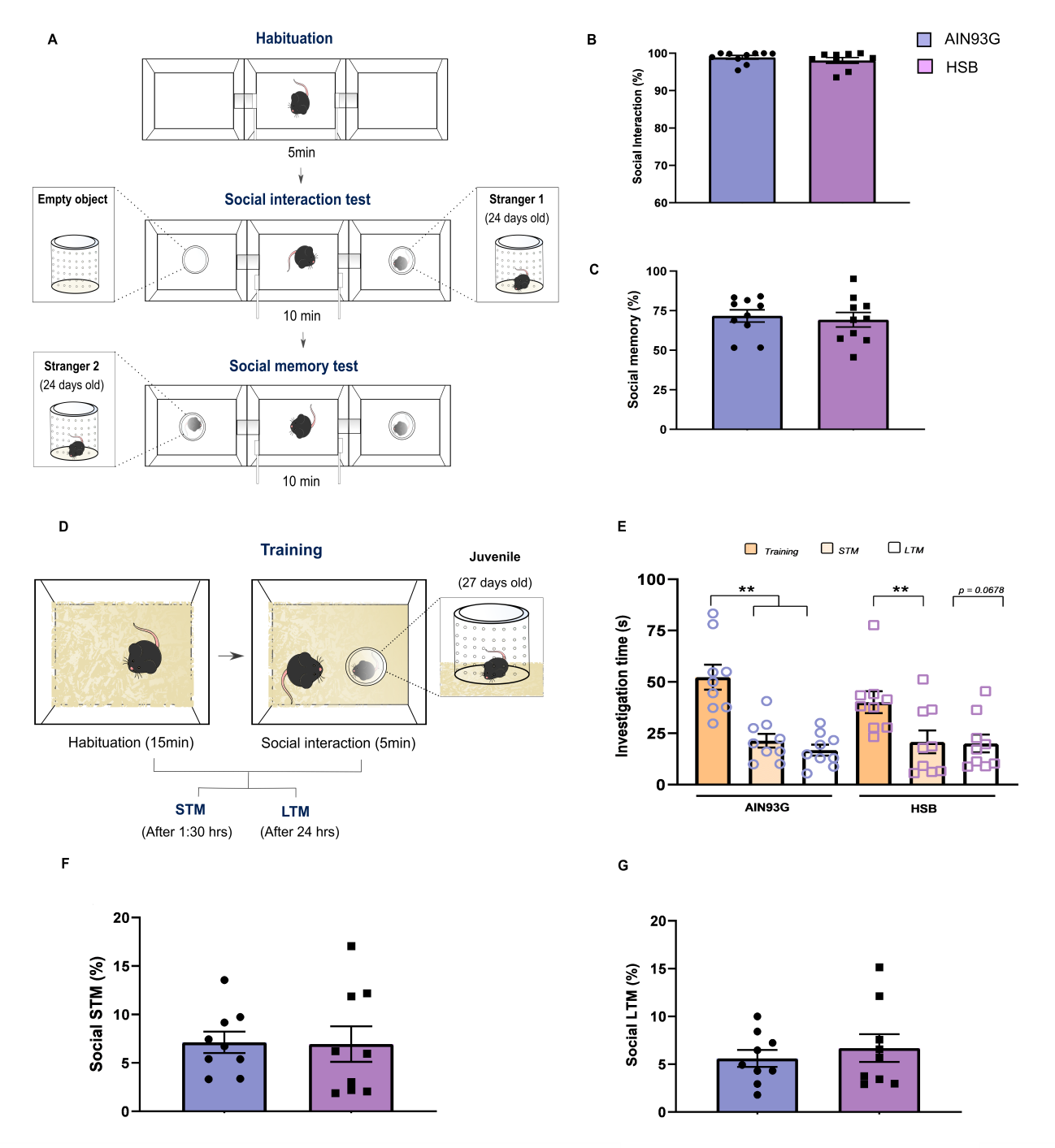
**

**Figure S4. Chronic consumption of HSB does not alter social interaction and social memory. (A)** Schematic representation of three-chamber social paradigm test. **(B-C)** Representation of general social interaction and social memory index, respectively, obtained from a three-chamber social paradigm. **(C)** In the one-chamber social memory test, short and long-term social memory (STM and LTM) were respectively evaluated after 1:30 and 24 hours after the training session. **(D)** Computation of raw interaction time with juvenile mice in training and experimental sessions. **(E-F)** Calculation of STM and LTM percentual. Error bars represent the mean ± SEM; n =9-10. Mann-Whitney test (**B**), Unpaired two tail T-student test (**C, E,** and **F**), and One-way repeated measures-ANOVA, followed by Tukey post-test (**D**). **p <0.01.

Table S1- Primers sequences utilized for RT-PCR

| **Targets** | **Sequence** |
| --- | --- |
| **GLUR2 (AMPA2)-F** | *ATGCGACCTGACCTCAAAGG* |
| **GLUR2 (AMPA2)-R** | *AGCAGAATCCAGCACAGCTT* |
| **NR1(NMDA) - F** | *ACTCCCAACGACCACTTCAC* |
| **NR1(NMDA) – R** | *GTAGACGCGCATCATCTCAA* |
| **NR2A (NMDA) - F** | *TACTCCAGCGCTGAACATTG* |
| **NR2A (NMDA) – R** | *CATGCGTGATGAGGCTCTTA* |
| **NR2B (NMDA) – F** | *GTGAGAGCTCCTTTGCCAAC* |
| **NR2B (NMDA) - R** | *ATGAAAGGGTTTTGCGTGAC* |
| **mGluR5 - F** | *TGTAACTCAAGACCTGAATCTATGG* |
| **mGluR5- R** | *GCTGGGCCAACTGAACTTTA* |
| **Slc1a2 (GLT-1)-F** | *ATTGGTGCAGCCAGTATTCC* |
| **Slc1a2 (GLT-1)-R** | *CCAGCTCAGACTTGGAAAGG* |
| **Cx3CL1-F** | *CGACAAGATGACCTCACGAA* |
| **Cx3CL1-R** | *CTGTGTCGTCTCCAGGACAA* |
| **Cx3CR1-F** | *TGCCTTCTTCCTCTTCTGGA* |
| **Cx3CR1-R** | *TAAAGGGGTTGAGGCAACAG* |
| **Claudin-5-F** | *GTTAAGGCACGGGTAGCACT* |
| **Claudin-5-R** | *GACAACGATGTTGGCGAACC* |
| **ZO-1-F** | *CAACAGGTACAGGCCAGAGG* |
| **ZO-1-R** | *ACTGCTTGGGCTCAGATGAC* |
| **Occludin-F** | *ATGTCCGGCCGATGCTCTC* |
| **Occludin-R** | *TTTGGCTGCTCTTGGGTCTGTAT* |
| **IL-1β- F** | *GGGCCTCAAAGGAAAGAATC* |
| **IL-1β- R** | *TACCAGTTGGGGAACTCTGC* |
| **IL-6- F** | *AACGCTACACACTGCATCTTGG* |
| **IL-6- R** | *GCCGTGGCAGTAACAGCC* |
| **TNF-α- F** | *TTCTGGCTCAAAAAGAGAATT* |
| **TNF-α- R** | *TGGTGGTCTTGTTGCTTAAGG* |
| **INF-ɣ- F** | *TCAAGTGGCGATGTGGAAGAA* |
| **INF-ɣ- R** | *TGGCTCTGCAGATTTTCATG* |
| **SYP-F** | *CAGTGTTCGCTTTCATGTGG* |
| **SYP-R** | *ACAGGGTCCCTCAGTTCCTT* |
| **DLG4 -F** | *TCTGTGCGAGAGGTAGCAGA* |
| **DLG4 -R** | *AAGCACTCCGTGAACTCCTG* |
| **ARC-F** | *GCTGAAGCAGCAGACCTGA* |
| **ARC-R** | *TTCACTGGTATGAATCACTGCTG* |
| **Rpl32-R** | *GCTGCCATCTGTTTTACGG* |
| **Rpl32-R** | *TGACTGGTGCCTGATGAACT* |
| **GAPDH - F** | *GGTGAAGGTCGGTGTGAACT* |
| **GAPDH - R** | *CTCGCTCCTGGAAGATGGTG* |
| **HPRT- F** | *GTTAAGCAGTACAGCCCCAAA* |
| **HPRT- R** | *AGGGCATATCCAACAACAAACTT* |
